# Intratumoral heterogeneity in a minority of ovarian low-grade serous carcinomas

**DOI:** 10.1186/1471-2407-14-982

**Published:** 2014-12-18

**Authors:** Alicia A Tone, Melissa K McConechy, Winnie Yang, Jiarui Ding, Stephen Yip, Esther Kong, Kwong-Kwok Wong, David M Gershenson, Helen Mackay, Sohrab Shah, Blake Gilks, Anna V Tinker, Blaise Clarke, Jessica N McAlpine, David Huntsman

**Affiliations:** Department of Pathology and Laboratory Medicine, University of British Columbia, Vancouver, BC Canada; BC Cancer Agency, Room 3427, 600 West 10th Avenue, Vancouver, BC Canada; Division of Gynecologic Oncology, Princess Margaret Cancer Centre, Toronto, ON Canada; Department of Computer Science, University of British Columbia, Vancouver, BC Canada; Department of Gynecologic Oncology & Reproductive Medicine, University of Texas MD Anderson Cancer Center, Houston, TX USA; Division of Medical Oncology and Hematology, Princess Margaret Hospital, Toronto, ON Canada; Laboratory Medicine and Pathobiology, University of Toronto, Toronto, ON Canada; Obstetrics and Gynecology, University of British Columbia, Vancouver, BC Canada

**Keywords:** Heterogeneity, Low-grade cancer, Ovarian serous carcinoma, Mutation, KRAS, BRAF, NRAS, SMAD4

## Abstract

**Background:**

Ovarian low-grade serous carcinoma (LGSC) has fewer mutations than ovarian high-grade serous carcinoma (HGSC) and a less aggressive clinical course. However, an overwhelming majority of LGSC patients do not respond to conventional chemotherapy resulting in a poor long-term prognosis comparable to women diagnosed with HGSC. *KRAS* and *BRAF* mutations are common in LGSC, leading to clinical trials targeting the MAPK pathway. We assessed the stability of targetable somatic mutations over space and/or time in LGSC, with a view to inform stratified treatment strategies and clinical trial design.

**Methods:**

Eleven LGSC cases with primary and recurrent paired samples were identified (stage IIB-IV). Tumor DNA was isolated from 1–4 formalin-fixed paraffin-embedded tumor blocks from both the primary and recurrence (n = 37 tumor and n = 7 normal samples). Mutational analysis was performed using the Ion Torrent AmpliSeq^TM^ Cancer Panel, with targeted validation using Fluidigm-MiSeq, Sanger sequencing and/or Raindance Raindrop digital PCR.

**Results:**

*KRAS* (3/11), *BRAF* (2/11) and/or *NRAS* (1/11) mutations were identified in five unique cases. A novel, non-synonymous mutation in *SMAD4* was observed in one case. No somatic mutations were detected in the remaining six cases. In two cases with a single matched primary and recurrent sample, two *KRAS* hotspot mutations (G12V, G12R) were both stable over time. In three cases with multiple samplings from both the primary and recurrent surgery some mutations (*NRAS Q61R, BRAF V600E, SMAD4 R361G*) were stable across all samples, while others (*KRAS G12V, BRAF G469V*) were unstable.

**Conclusions:**

Overall, the majority of cases with detectable somatic mutations showed mutational stability over space and time while one of five cases showed both temporal and spatial mutational instability in presumed drivers of disease. Investigation of additional cases is required to confirm whether mutational heterogeneity in a minority of LGSC is a general phenomenon that should be factored into the design of clinical trials and stratified treatment for this patient population.

**Electronic supplementary material:**

The online version of this article (doi:10.1186/1471-2407-14-982) contains supplementary material, which is available to authorized users.

## Background

In comparison to the more commonly occurring high-grade serous carcinomas (HGSC), ovarian low-grade serous carcinomas (LGSC) are characterized by a younger age at onset, lower mitotic rate and longer median overall survival [1–6]. Whereas the vast majority (80%) of patients with HGSC are responsive to platinum-based chemotherapy, patients with LGSC are highly resistant to treatment in the neoadjuvant, adjuvant and recurrent setting, with response rates of 4-5% [1, 7, 8]. Women diagnosed with LGSC typically experience multiple recurrences over a protracted clinical course before ultimately dying of their disease, with an associated 10-year survival rate of <50% [2]. This suggests that despite having a less aggressive clinical course, women with LGSC have a poor long-term prognosis similar to HGSC patients; this is highlighted by a recent study reporting a similar hazard ratio for death in LGSC and HGSC patients with measurable residual disease after adjusting for additional variables [9].

In an effort to identify potential molecular targets, limited mutational studies in primary or recurrent LGSC samples have revealed an overall low mutation frequency, with exome sequencing by Jones *et al.* showing an average of 10 validated somatic mutations (or 7.5 somatic non-synonymous or splice site mutations) per tumor [10]. The mitogen-activated kinase (MAPK) pathway is most frequently mutated [11], with 19-35% of cases containing a *KRAS* mutation and 2-33% containing a *BRAF* mutation [3, 10, 12–14]. *KRAS* and *BRAF* mutations are also frequently detected in serous borderline tumors (SBT), the histologic precursor to invasive LGSC [5, 6, 11, 15–17].

The prevalence of *KRAS/BRAF* mutations in LGSC has resulted in clinical trials of inhibitors of MAP kinase kinase (MEK1/2), which lies immediately downstream of BRAF and upstream of ERK1/2 in the MAPK pathway [18, 19]. Previous studies have reported profound growth inhibition and apoptosis in ovarian cancer cells with mutated but not wildtype *KRAS* or *BRAF* upon treatment with CI-1040 [20] in tissue culture and xenograft studies [19, 21], suggesting that mutation status predicts sensitivity to MEK inhibition. A recent phase II study of selumetinib, another small molecular inhibitor of MEK1/2, in women with recurrent ovarian/peritoneal LGSC has shown an objective 15% response rate despite heavy pre-treatment; however patient response does not appear to be correlated with *KRAS/BRAF* mutation status [18]. The mutation status of the patients in this trial was based solely on a single sample of LGSC; most were obtained from the primary tumor and a small percentage were obtained from the recurrent tumor. In this study we aimed to assess the stability of targetable mutations over space and/or time by targeted sequence analysis of one or more tumor samples from both the primary and recurrence, to inform future clinical trial design. Herein we report our findings of mutational stability in the majority of cases, as well as remarkable instability in one case of ovarian LGSC, in presumed drivers of disease *KRAS* and *BRAF*. If validated in more cases this could impact clinical trial design for this patient population in the future.

## Methods

### Study cases

A total of 11 cases of LGSC with matched primary and recurrent samples available were identified from the University Health Network in Toronto, Ontario (“UHN”, n = 3), MD Anderson Cancer Center in Houston, Texas (“MDACC”, n = 3) and the BC Cancer Agency in Vancouver, British Columbia (“BCCA”, n = 5). The stage breakdown included: IIB (n = 1), IIIB (n = 3), IIIC (n = 6) and IV (n = 1). Research ethics approval was obtained from each site (UBC BCCA Research Ethics Board, University Health Network Research Ethics Board and The University of Texas MD Anderson Cancer Center Institutional Review Board). All patients provided written informed consent to have their tissue samples used for research purposes, including genomic studies. Written informed consent was obtained from every patient for publication of the specific clinical details included within this research article and any accompanying images. However, potentially identifying information such as date of diagnosis have been removed to protect privacy. Upon inclusion in the study, all formalin-fixed paraffin-embedded (FFPE) sections chosen for sequence analysis were subjected to secondary pathologic review (B.G.). Two of eleven cases originally presented as a SBT, and recurred as invasive LGSC. Nine patients received adjuvant treatment following diagnosis, including six treated with combined carboplatin/paclitaxel.

A total of 37 tumor samples (from either FFPE blocks [BCCA] or unstained sections [MDACC/UHN]) were included for analysis. At least one sampling from both the primary and recurrent setting were included for each case, with H&E-guided macrodissection used to isolate tumor from adjacent stromal cells. Tumor cellularity achieved following macrodissection was estimated at a median of 80% (range 50-95%) and was comparable among samples obtained from the same case. Normal samples were available for 7 cases (6 matched normal tissue, 1 buffy coat). We were also able to obtain a fresh blood sample from one BCCA study patient for extraction of circulating tumor DNA (“ctDNA”). Summary information for all study cases is included in Table 1, with more detailed information on tumor sites and normal samples used in Additional file 1 and case images in Additional files 2, 3, 4, 5, 6, 7, 8, 9, 10, 11 and 12.

**Table 1:** **Summary of study cases**

Case	Source	Age	# Tumor samples	Stage	Primary tumor samples	Intervening Tx/s	Recurrent samples (***Time to recurrence)	***Time to/Status last followup
LGSC-2	UHN	57	2	IIIC	2-P*	Carboplatin/paclitaxel	2-R (46 mo)	86 mo / AWD
LGSC-3	UHN	51	2	IIIC	3-P*	Carboplatin/paclitaxel	3-R (17 mo)	19 mo / AWD
LGSC-4	UHN	66	3	IIIB	4-P	Carboplatin	4-R1 (25 mo), 4-R2 (45 mo)	60 mo / DOD
LGSC-5	MDACC	51	2	IIIC	5-P	Carboplatin/paclitaxel, letrozole	5-R (37 mo)	53 mo / DOD
LGSC-6	MDACC	41	2	IIIC	6-P	Carboplatin/paclitaxel	6-R (24 mo)	87 mo / DOD
LGSC-8	MDACC	33	2	IIIC	8-P	Cisplatin/cyclophos-phamide	8-R (7 mo)	12 mo / DOD
LGSC-9**	BCCA	51	6	IIIB	9-P1, P2, P3*	No treatment	9-R1, R2, R3 (100 mo)	141 mo / DOD
LGSC-10	BCCA	57	8	IV	10-P1, P2, P3, P4*	Carboplatin/paclitaxel, radiation	10-R1, R2, R3, R4 (45 mo)	62 mo / DOD
LGSC-11**	BCCA	62	2	IIIC	11-P*	No treatment	11-R (156 mo)	180 mo / DOD
LGSC-12	BCCA	57	6	IIB	12-P1, P2, P3, P4*	Etoposide, tamoxifen, anastrozole	12-R1, R2 (18 mo)	53 mo / DOD
LGSC-13	BCCA	58	2	IIIB	13-P*	Carboplatin/paclitaxel	13-R (46 mo)	59 mo / DOD
**Mean (Total)**		**53.1**	**3.4 (37)**					

*normal sample also available; **initial diagnosis of SBT; ***time in months since diagnosis.

*Abbreviations:* AWD = alive with disease, DOD = dead of disease.

### DNA extraction

Tumor and normal DNA was extracted from FFPE blocks/unstained sections (see Additional file 13 for supplemental methods). For extraction of ctDNA, whole blood was collected in EDTA tubes then centrifuged at 2,500 rpm for 15 min. Plasma was then stored at -80C in 1-2 mL aliquots, followed by extraction of plasma ctDNA using the Qiagen Circulating Nucleic Acid kit as per manufacturer’s protocol. ctDNA was then eluted in 30uL of Buffer AVE.

### Ion torrent AmpliSeq cancer hotspot sequencing

The Ion Torrent AmpliSeq^TM^ Cancer Hotspot Panel Version 1 (Life Technologies, Grand Island/NY/USA) [22] was used to prepare sequencing libraries from all tumor DNA, normal DNA and plasma ctDNA as per manufacturer’s protocols (see Additional file 13 for methodological details and Additional file 14 for a comprehensive list of genes and mutations included on the AmpliSeq panel). A list of predicted variants was generated for each sample using the built-in AmpliSeq Cancer Variant Caller following each run. Upon completion of all samples, we performed a separate bioinformatics analysis. Sequence reads were aligned to the human reference sequence (UCSC hg19) using the BWA SW algorithm (v0.6.1) [23] with default parameters. Single nucleotide variants (SNV) were called using mutationSeq [24], a feature-based method to filter out technical artifacts. We used a variant probability threshold of 0.5 to nominate SNVs. Variants predicted in the pooled normal data were considered as germline mutations and were removed.

Variants were selected for targeted validation by MiSeq in all samples according to the following selection criteria: [1] either somatic (variant not found in corresponding normal sample if available) or predicted somatic (for cases with no corresponding normal – variant not found in dbSNP or in normal samples from other cases), [2] non-synonymous and [3] predicted functional impact according to MutationAssessor [25], a method to predict the functional impact of missense mutations on protein products based on evolutionary conservation of amino acid residues in multi-sequence alignment of homologous protein sequences.

### Fluidigm-MiSeq targeted sequencing validation

Variants identified by Ion Torrent AmpliSeq sequencing results were verified by validation sequencing using the Fluidigm 48X48 AccessArray amplification (Fluidigm, San Francisco/CA/USA) coupled with the Illumina MiSeq personal sequencer (Illumina Inc, San Diego/CA/USA) (see Additional file 13). Sequence reads were aligned using the mem algorithm of BWA v0.7.4 [26] to a reference database containing only the targeted loci. We inferred the presence/absence of the targeted variants using a Binomial exact test. In the context of this analysis, a somatic mutation was considered to be “validated” if: [1] both tumor and normal data had a minimum of 50 reads covering the targeted position, [2] the Binomial exact test result (Benjamini Hochberg adjusted p-value) for the tumor was <0.01, [3] the Binomial exact test result (Benjamini Hochberg adjusted p-value) for the normal was > =0.01, and [4] the proportion of reads indicating the variant in the tumor was ≥5%. For the cases without a normal control, the validated variants also shown in the pooled normal data were considered as germline mutations and were removed. All mutations were visually confirmed using the Integrative Genomics Viewer (Broad Institute, Cambridge/MA/USA).

### Sanger sequencing

Sanger sequencing was used to confirm select high allelic fraction mutations, using methods previously described [27]. Primer sequences are listed in Additional file 15.

### Raindance raindrop digital PCR assay

Custom TaqMan SNP Genotyping assays (Life Technologies, CA/USA) were used as primer/probes (40X) to confirm low allelic fraction mutations using the Raindance Raindrop digital PCR assay (Raindance, Billerica/MA/USA). Sequences for primers are shown in Additional file 16. Digital PCR assays were performed as per manufacturer’s protocols (see Additional file 13).

### Definition of true positive mutations

Only those mutations that were detected by at least two independent technologies were considered “true positives” and were included in intra-patient comparisons.

## Results

### Overall mutational landscape of LGSC study cases

**Figure 1:**
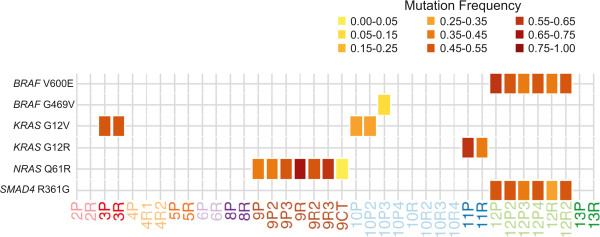
**Average allele fraction of confirmed somatic mutations by ion torrent and MiSeq.** The presence of a specific mutation (listed on left) in a specific tumor sample (listed at bottom) is indicated by a colored box in the corresponding position, with the shade of the box reflecting the average allele fraction as detected by Ion Torrent and MiSeq. Corresponding normal samples are not shown, as these were all negative for the described mutations.

Following initial screening by Ion Torrent and validation by MiSeq, Sanger and/or digital PCR, very few “true positive” mutations were observed overall, with only 7 mutations detected among 5 of 11 cases. No somatic mutations were observed in cases LGSC-2, LGSC-4, LGSC-5 and LGSC-13, whereas predicted mutations in cases LGSC-6 and LGSC-8 were only detected by one of the sequencing platforms used. True positives included *KRAS* mutations in 3 cases (n = 2 G12V and n = 1 G12R), *BRAF* mutations in 2 cases (n = 1 V600E and n = 1 G469V), and *NRAS* (Q61R) and *SMAD4* (R361G) mutations in one case each. The average allelic fraction of each of these mutations in individual samples as determined by Ion Torrent and MiSeq is shown in Figure 1 (see Additional file 17 and Additional file 18 for data from each platform). Mutational patterns over time/space will be discussed for individual cases in the following sections.

### Mutational stability over time

**Figure 2:**
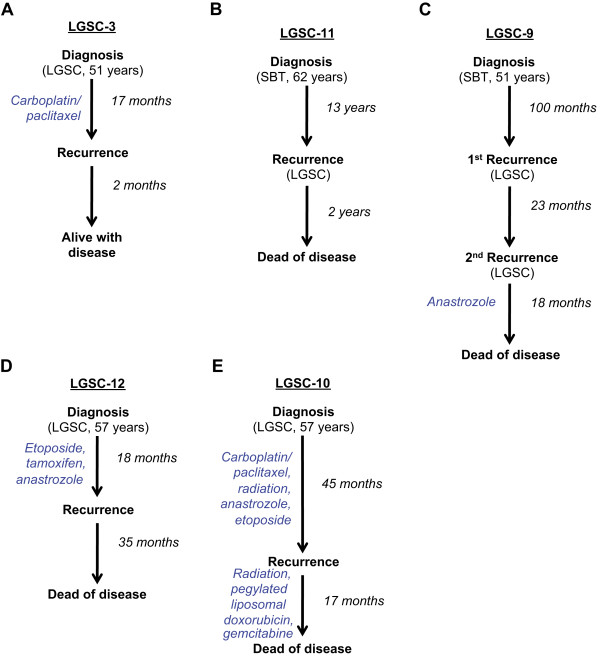
**Overview of clinical course for patients with true positive mutations.** The clinical course for LGSC-3 **(A)**, LGSC-11 **(B)**, LGSC-9 **(C)**, LGSC-12 **(D)** and LGSC-10 **(E)** are shown, with treatment at each step displayed on the left and time indicated on the right.

Two cases with one primary and recurrent sample each (LGSC-3 and LGSC-11) were used to assess temporal stability of confirmed somatic mutations (see Figure 2A-B for overview of clinical course).

**LGSC-3** is from a 51 year old patient diagnosed with bilateral ovarian LGSC with extensive extra-ovarian involvement, stage IIIC (described in Additional file 1). This patient received adjuvant carboplatin/paclitaxel prior to disease recurrence 17 months after primary diagnosis. At last follow-up (19 months post-diagnosis) the patient was alive with disease. Sequencing analysis discovered a *KRAS* hotspot mutation (chr12:25,398,284C > A, G12V) at a similar allelic fraction of ~50% (range 48-53%) in the primary and recurrent samples, suggesting that this was a stable feature in this tumor (see Additional file 19 for confirmation by Sanger).

**LGSC-11** is from a 62 year old patient diagnosed with stage IIIC SBT of the left ovary, with ovarian surface involvement and non-invasive implants. This patient received no additional treatment, recurred with metastatic LGSC 13 years later and died of disease 15 years post-diagnosis. The tumor was found to have a *KRAS* hotspot mutation (chr12:25,398,285C > G, G12R) at a similar allelic fraction in the primary (SBT, 57%) and recurrent (LGSC, 44%) sample by both Ion Torrent and MiSeq.

### Mutational stability over space and time

Multiple samplings from both the primary and recurrent setting from three cases (LGSC-9, LGSC-10 and LGSC-12) were used to assess the spatial and temporal stability of features (see Figure 2C-E for overview of clinical course).

**Figure 3:**
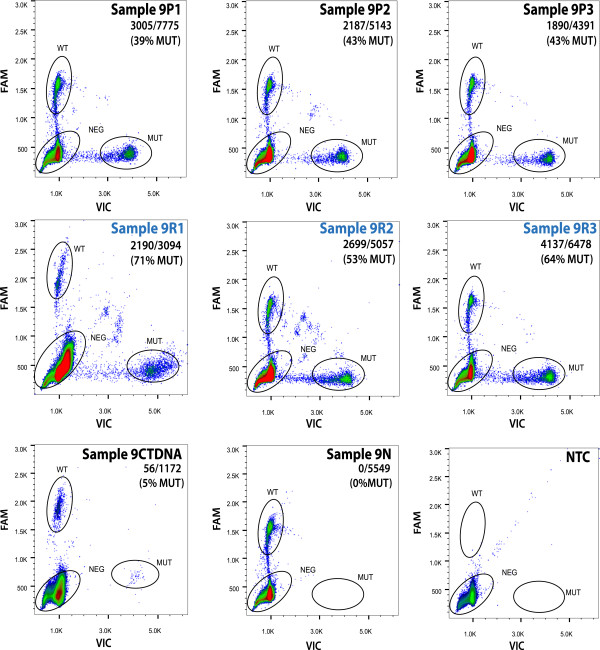
**Stability of** ***nras*** **q61r mutation in multiple tumor samplings over space and time and circulating tumor DNA.** Detection of the *NRAS* Q61R mutation in tumor samples from the original SBT (Sample 9P1-9P3, top panels) and first LGSC recurrence (Sample 9R1-9R3, middle panels) by the Raindance Raindrop digital PCR assay are shown. Mutation status was also determined in the ctDNA sample obtained following the second LGSC recurrence (Sample 9CTDNA), corresponding normal (Sample 9 N) and non template control (NTC) (bottom panels). The wild type ('WT’) and mutant ('MUT’) population are circled in each panel, with the % MUT indicated in the top right corner (MUT drops/total of WT + MUT droplets). Consistent with Ion Torrent and MiSeq, the *NRAS* Q61R mutation was observed in all 6 tumor samples and the ctDNA sample, and was not detected in the corresponding normal.

**LGSC-9** is from a 51 year old patient diagnosed with stage IIIB SBT of the right ovary with non-invasive implants. No additional treatment was given after primary surgery. More than 8 years (100 months) following initial diagnosis, there was tumor recurrence involving the ovary and rectosigmoid, demonstrating malignant transformation to LGSC with borderline features. This was treated by complete surgical resection. A second recurrence (sigmoid mass) of LGSC occurred 23 months later. At this time she was treated with anastrozole (a non-steroidal aromatase-inhibitor [28]), and died of disease 141 months following initial diagnosis. Sequencing analysis revealed a somatic, non-synonymous mutation in *NRAS* (chr1:115,256,529 T > C, Q61R) at a comparable allele fraction (mean of 50%, range 40-73%) in all six tumor samples assessed, including 3 samplings from the original SBT and 3 from the first recurrence of LGSC (2 from rectosigmoid and 1 from the left pelvic sidewall). The same mutation was also observed at a lower fraction (5%) in a fresh ctDNA sample obtained following the second recurrence. The stability of this mutation among all 7 samples was confirmed by digital PCR (Figure 3/Additional file 20).

**LGSC-12** is from a patient diagnosed with stage IIB LGSC at the age of 57. Her disease was distributed throughout the pelvis with implants on the rectosigmoid colon. Following diagnosis, this patient was treated with etoposide (topoisomerase inhibitor), tamoxifen (estrogen receptor inhibitor) and anastrozole (non-steroidal aromatase inhibitor), before recurring 18 months later with LGSC involving the abdominal wall. She died of disease 53 months following her original LGSC diagnosis. Of note, this patient had a documented history of SBT 36 years prior to her diagnosis with LGSC; however tissue samples were not available for analysis. Sequencing of 4 primary LGSC samples (including 2 from the pelvic tumor, 1 from the rectosigmoid tumor and 1 from a peri-aortic tumor nodule) and 2 recurrent LGSC samples (both from the abdominal wall tumor) revealed somatic non-synonymous mutations in both *BRAF* (chr7:140,453,136A > T, V600E) and *SMAD4* (chr18:48,591,918C > G, R361G). Both of these mutations had an allelic fraction of 31-55% in all samples (*BRAF* median 51%, range 37-55%; *SMAD4* median 49%, range 31-51%), suggesting that they were both stable over space and time (see Additional file 19 for confirmation by Sanger in select samples).

**Figure 4:**
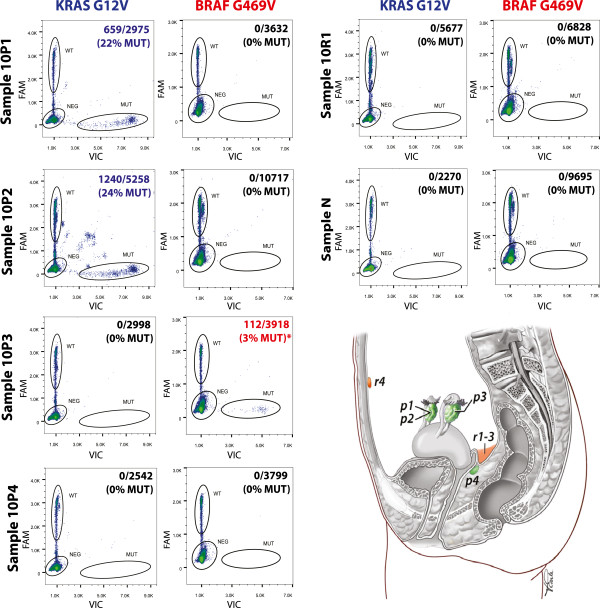
**Instability of** ***KRAS*** **G12V AND** ***BRAF*** **G469V mutations over both space and time.** Raindance Raindrop digital PCR was used to confirm *KRAS* and *BRAF* mutation status in all 8 tumor samples and the corresponding normal, with the four samples from the primary surgery (Sample 10P1-10P4) shown on the left and a representative sample from the recurrent surgery (Sample 10R1) and the corresponding normal (Sample 10 N) shown on the right. The relative location of each sample in the patient is shown in the bottom right, with those from the primary surgery colored in green and those from the recurrent surgery colored in orange (courtesy of Vicky Earle, UBC graphics). Similar to Figure 3, the wild type ('WT’) and mutant ('MUT’) population are circled in each panel, with the % MUT indicated in the top right corner (MUT drops/total of WT + MUT droplets). The *KRAS* G12V mutation was detected in Samples 10P1 and 10P2, while the *BRAF* G469V mutation was exclusively detected in Sample 10P3. All remaining samples were negative for *KRAS* G12V, *BRAF* G469V and *NRAS* Q61R (not shown).

**LGSC-10** is from a patient diagnosed with bilateral ovarian LGSC with extensive extra-ovarian involvement (stage IV) at the age of 57. Adjuvant treatment included 6 cycles of carboplatin/paclitaxel, radiation, anastrozole and etoposide. This patient recurred with LGSC 45 months later at which point she was treated with radiation, liposomal doxorubicin chemotherapy and gemcitabine before dying of her disease 62 months following initial diagnosis. Unlike cases LGSC-9 and LGSC-12, sequencing of 4 primary and 4 recurrent samples revealed extensive mutational variability. As shown by digital PCR in Figure 4 (and Additional file 20), 2 of 4 specimens from the primary setting, both from the right ovary, contained a *KRAS* G12V hotspot mutation (22-31% allele fraction), while the specimen from the left ovary contained a low level (3-7%) *BRAF* mutation (chr7:140,481,402C > A, G469V). Neither of these mutations were detected in the remaining specimen from the primary surgery (vaginal septal tumor) or any of the recurrent samples (including 3 from a right lower quadrant subcutaneous nodule and 1 from an umbilical margin large nodule). This was not a reflection of tumor purity, as all mutation-negative specimens had comparable tumor cellularity by histopathologic assessment (80-95%) and identical allele fractions of common SNPs in *FGFR3* and *PDGFRA* (≥99%, data not shown).

### Overall trends in mutational stability

As shown in Table 2, four of five cases with true positive mutations were stable over time and/or space, including two cases that originally presented as SBT and recurred as an invasive LGSC. In contrast, one case showed instability of *KRAS* and *BRAF* over both space and time. Overall, mutations in *NRAS* and *SMAD4* were stable in one case each, while genes mutated in more than one study case (*KRAS* and *BRAF*) showed different patterns of stability/instability for distinct variants (*BRAF* V600E vs. G469V, *KRAS* G12R vs. G12V) and even for the same variant (*KRAS* G12V).

**Table 2:** Overall trends in stability over time and space for confirmed somatic mutations in LGSC*

	Time		Time and Space		
	LGSC-3	LGSC-11	LGSC-9	LGSC-10	LGSC-12
BRAF G469V				Unstable	
BRAF V600E					Stable
KRAS G12R		Stable			
KRAS G12V	Stable			Unstable	
NRAS Q61R			Stable		
SMAD47 R361G					Stable

*Only those mutations observed by two independent technologies (true positives) included.

Note: not all cases included in table as no confirmed somatic mutations in LGSC-2, -4, -5 or -13; mutations in LGSC-6 and -8 only observed by either Ion Torrent or MiSeq.

## Discussion

Among the 11 cases of LGSC sequenced in our study, only 7 confirmed somatic mutations were identified in 5 cases from a targeted hotspot panel of 46 cancer-associated genes. This low mutation rate is consistent with the detection of only 10 mutations per tumor by exome sequencing by Jones *et al.*[10], and further suggests that few mutational events are required to achieve malignancy. The frequency of mutations in LGSC is much lower than in other subtypes of ovarian carcinoma such as HGSC (n = 61 mutations/tumor by exome sequencing) [29] and clear cell carcinoma (n = 34 mutations/tumor by exome sequencing) [30]. This likely suggests that: [1] there is limited replication of precursor cells prior to initiation of tumorigenesis, [2] there are few bottlenecks once initiation occurs, and [3] the ratio of driver to passenger mutations should be higher than in other tumor types [10]. Consequently, targeted agents would likely be particularly effective in women with LGSC if key mutations are shown to be stable.

The most commonly reported drivers in LGSC are *KRAS* and *BRAF*. We detected a *KRAS* mutation in three patients (including two stage IIIC and one stage IV) and a *BRAF* mutation in two patients (including one stage IIB and one stage IV). Previous studies have reported conflicting findings with respect to mutation of *KRAS/BRAF* and disease stage, with the Jones study [10] detecting *KRAS* or *BRAF* mutations in 4/13 (31%) and 3/13 (23%) of stage III LGSC patients respectively. Additional studies report *BRAF* mutations in only 3% [12] and 5% [13] of advanced stage LGSC. Grisham and Wong both reported that women with mutations in *KRAS* and/or *BRAF*[12, 13] experience a more favorable outcome than women without these mutations. This positive prognostic effect appears to be dominated by *BRAF* V600E mutations, with a lower incidence of stage III-IV disease, enrichment for SBT rather than invasive LGSC and reduced requirement for systemic treatment among women with this mutation [12, 13]. Possible explanations include reports that SBTs from women with *BRAF* mutations over-express genes with cell growth inhibitory effects [12] or that activating *BRAF* mutations induce cellular senescence and prevent progression to LGSC [12, 31–33]. In our study we observed a trend for increased mean overall survival in study patients with a MAPK pathway mutation *(KRAS, BRAF, NRAS)* compared to patients with wildtype status (92 months vs. 60 months respectively; p = 0.23); however, this difference in outcome was largely influenced by the two cases originally presenting as a SBT (143 and 183 months) and disappeared when these cases were excluded from the analysis.

The mutational status of *NRAS*, member of the MAPK pathway, showed stability over multiple different tumor sites and over a span of 8 years between original diagnosis with SBT and recurrence with an invasive LGSC (case LGSC-9). The presence of this stable feature at a low level in plasma ctDNA, obtained following a second recurrence of LGSC, also clearly highlights the potential utility of this source for disease monitoring (i.e. tumor response, persistence or recurrence).

*SMAD4* mutational status in case LGSC-12 was also consistent among 6 tumor samples from 4 different sites in the primary and recurrence, and despite multiple treatment cycles. Although found to be unstable in another case, all samples from LGSC-12 also contained a *BRAF* mutation at a similar allelic fraction. The observed *SMAD4* mutation (chr18:48,591,918C > G, R361G) is at a highly conserved genomic position among placental mammals, and is situated within the C-terminus MH2 domain of the SMAD4 protein. This domain mediates protein-protein interactions and provides functional specificity and selectivity. It was previously reported as the most frequent target of *SMAD4* missense mutations in human tumors, with a mutational hotspot corresponding to codons 330–370 [34]. Lassus *et al.* reported allelic loss at one or more loci at 18q12.3-q23 in 59% of ovarian serous carcinomas (or 7.1% of grade 1 tumors), with lost or weak expression of SMAD4 protein in a subset of these tumors [35]. Mutations in *SMAD4* have been reported to frequently co-exist with *KRAS* mutations in colorectal cancer [36], and studies in pancreatic cancer suggest that wildtype SMAD4 blocks progression of *KRAS* G12D-initiated tumors [37]. In addition, mutation of *KRAS, NRAS* and *BRAF*[38–46], and loss of functional SMAD4 [47], have all been reported to predict resistance to anti-EGFR therapy. Unfortunately we were unable to assess the impact of the *SMAD4* R361G mutation on protein expression by IHC in our samples, therefore we cannot comment on the utility of *SMAD4* mutation status as a predictive marker in women with LGSC without further study.

In contrast to *NRAS* and *SMAD4*, mutations in *KRAS* and *BRAF* were not stable in one patient (LGSC-10) in our study, despite traditionally being thought of as 'drivers’ of tumorigenesis. This is akin to our recent observation that mutations in other key 'drivers’ *PIK3CA* and *CTNNB1* are only present in a subset of ovarian HGSC samples from the same patient [48]. These examples clearly defy the concept of oncogene addiction, which posits that the growth and survival of a tumor is dependent on a single dominant oncogene [49, 50]. Our findings in LGSC-10 suggest that even at the time of primary diagnosis three distinct tumors/clones were present (i.e. *KRAS*-mutation positive, *BRAF*-mutation positive and *KRAS/BRAF*-mutation negative). As neither *KRAS* nor *BRAF* were mutated in any of the recurrent samples, a different, as yet unidentified, dominant gene or pathway in the *KRAS/BRAF*-negative population was likely driving disease recurrence. One possibility is that we have missed a mutation in gene/s either directly or indirectly involved in the MAPK pathway that is not included on the targeted panel used to screen our samples. The *KRAS* and *BRAF* mutations were detected at an allelic fraction of 22-31% in the right ovary and 3-7% in the left ovary respectively, hence the clonal population containing an undetected driver mutation could have already been present in some or all of the tumor samples at primary debulking; expansion/recurrence of this population could then explain the absence of mutant *KRAS/BRAF* in the recurrent setting. In addition, mutations such as those in *KRAS* and *BRAF* that occur early in the development of SBT/LGSC [17] may not be required and/or advantageous for tumor maintenance once additional alterations are acquired. This phenomenon has previously been described in HGSC, in which secondary mutations in *BRCA1/2* restore protein function and result in acquired resistance to treatment [51]; however, reversion of both a *KRAS* and *BRAF* mutation in the current scenario seems highly unlikely.

Of potential interest, LGSC-10 was the only study case diagnosed with stage IV disease and the only patient treated with radiation after primary diagnosis. While the presence of mutational instability in the primary setting (prior to treatment) argues against a direct impact of radiation, the possibility of instability exclusively in stage IV LGSC is an intriguing one that requires more study. To date, limited studies have reported on either temporal or spatial instability of *BRAF/KRAS* mutations in SBT and LGSC. Instability in *KRAS* mutation status was recently described in a subset of matched SBT-LGSC pairs (2/5 cases discordant) [52] and matched SBT-peritoneal implant pairs (3/37 discordant for *KRAS*, while 14/14 concordant for *BRAF*) [53]. A recent study by Heublein *et al.*[54] also noted instability in *KRAS* and *BRAF* in 2/5 cases of bilateral SBT. In one case, a *KRAS* G12V mutation was detected in one ovary and a *BRAF* V600E mutation was detected in the contralateral ovary, while the other case contained a *KRAS* G12V and *BRAF* V600E mutation in one ovary and only a *KRAS* G12V mutation in the other ovary. This is consistent with our finding of spatial heterogeneity in the primary setting in LGSC-10. Unfortunately, a detailed breakdown of disease stage in cases with discordant vs. concordant sample pairs was not provided in any of these studies. Instability in *KRAS* has also been described for metastatic colorectal cancer [55, 56]. Bossard *et al.*[55] reported several patterns of heterogeneity in *KRAS* mutation status in 22% of 18 colorectal carcinomas studied. This included exclusive presence in the primary tumor or metastatic site, presence in some metastases but not others, varied status among different samplings from the same metastatic site, and presence in the recurrent but not primary setting. Similarly, Otsuka *et al.*[56] reported the presence of a *KRAS* mutation in metastatic sites but not the primary colorectal tumor in 1 of 9 patients studied; *BRAF* mutation status was concordant in all cases, in contrast to what we observed.

Our finding that mutations in genes such as *KRAS* or *BRAF* are not necessarily stable features could provide an alternative explanation, in some patients, for the lack of correlation between response to selumetinib and *KRAS/BRAF* mutation status observed by Farley *et al.*[18]. Targeted sequencing (i.e. codon 599 of *BRAF* and codons 12 and 13 of *KRAS*) using a single representative tumor sample from 34/52 (65%) patients revealed a *BRAF* and *KRAS* mutation in 2 (6%) and 14 (41%) cases respectively. A similar proportion of mutation positive vs. negative cases responded to selumetinib treatment, leading the authors to postulate that its activity may not depend on *BRAF/KRAS* mutational activation. Tissue used for mutational analysis was obtained from the primary tumor in 82% of sequenced cases, metastatic tumor in 6% and recurrent or persistent tumor in 12% of cases. It is therefore possible that targetable mutations detected in the primary tumor were not present in the metastatic or recurrent tumor, or vice versa, leading to altered treatment response. It is also possible that some of these patients had undetected mutations in *NRAS*, a stable feature in our study, which also predicts response to MEK inhibitors.

It is important to recognize the limitations of our study, most notably small sample size and use of a hotspot targeted gene panel. Firstly, the small number of cases used in this study (despite being a collaboration between three institutions) is illustrative of the challenge in identifying primary-recurrent pairs for a rare tumor type such as LGSC. Confirmation of our findings in a larger cohort of LGSC will therefore require participation by multiple institutions or establishment of a worldwide registry. Secondly, by limiting the sequencing discovery phase to a panel of hotspot mutations in 46 genes, it is highly likely that we have missed additional case-specific mutations in our study population. However, a closer look at the mutations discovered by Jones *et al.* through exome sequencing [10] revealed that only *KRAS* and *BRAF* were recurrently mutated in LGSC. This suggests that it is also unlikely that we have missed additional recurrent drivers of disease, although patient-specific drivers outside the normal patterns of LGSC may exist. Thirdly, we have not investigated potential alternative drivers of disease that may be important in cases without identified somatic mutations, such as copy number alterations, epigenetic changes or microRNAs. Singer *et al.*[57] previously reported a progressive increase in copy number alterations from SBT through to LGSC, most notably allelic imbalance of chromosomes 1p, 5q, 8p, 18q and 22q. This was confirmed by Kuo *et al.*[58] who reported an increased chromosomal instability index in LGSC relative to SBT, suggesting that amplifications, deletions and aneuploidy play a role in the malignant transformation of SBT. Hemizygous deletion of chromosome 1p36 was especially enriched in LGSC samples; this region contains the microRNA miR-34a, which was found to have an anti-proliferative and pro-apoptotic effect in an LGSC cell line [58]. Finally, several groups have reported on differential methylation patterns in SBT and LGSC [59–61], suggesting that methylation-induced transcriptional silencing of tumor suppressor genes may play an undefined role in malignant transformation and progression and response to systemic or targeted therapy.

## Conclusions

The extent of intratumoral heterogeneity in kidney, breast, leukemia and ovarian cancers has recently been described [48, 62–64]. Most papers have focused on high-grade cancers with many somatic mutations, and most of the mutations described have no immediate clinical relevance. Herein we show that, in a cancer type known to have a sparse mutational landscape [10], heterogeneity in targetable mutations can be observed. While the vast majority of evaluable cases contained mutations that were detected in all samples, one case showed remarkable instability in hotspot mutations of presumed drivers of disease, despite not receiving treatment that could have driven the specific evolution of (*KRAS/BRAF*) mutant clones. In addition, as we looked within a limited mutational space, the possibility remains that more underlying heterogeneity may be revealed in more cases with further study. Investigation of additional cases is required to confirm whether a consistent minority of LGSC cases show clinically relevant mutational heterogeneity; this would necessitate a change in clinical trial design with contemporary samplings of a cancer required to guide treatment decisions. Alternatively, if not found to be a general phenomenon upon further study, confirmation of mutational status in a single sample would be sufficient.

## Authors’ information

Alicia A. Tone, PhD

Scientific Associate II

Melissa K. McConechy, BSc

Doctoral Candidate

David Huntsman, MD, FRCPC, FCCMG

Dr. Chew Wei Memorial Professor of Gynaecologic Oncology

UBC Professor, Departments of Pathology & Lab Medicine and Obstetrics & Gynaecology UBC Director of OvCaRe, Vancouver General Hospital, BC Cancer Agency

UBC Medical Director, Centre for Translational and Applied Genomics, PHSA Laboratories

## Electronic supplementary material

Additional file 1: **“Additional Information on Study Samples”.** Provides more detailed information on pathologic diagnosis, DNA quantity and estimated cellularity. (XLSX 19 KB)

Additional file 2: **“LGSC-2 Case Images”.** LGSC-2 is from a patient diagnosed with bilateral ovarian LGSC (stage IIIC) at 57 years old (LGSC-2-P, top) and metastatic LGSC 46 months after primary diagnosis (LGSC-2-R, bottom; both 20X). (PDF 6 MB)

Additional file 3: **“LGSC-3 Case Images”.** LGSC-3 is from a patient diagnosed with bilateral ovarian LGSC (stage IIIC) at 51 years old (LGSC-3-P, top), and recurrent LGSC 17 months later (LGSC-3-R, bottom; both 20X). (PDF 5 MB)

Additional file 4: **“LGSC-4 Case Images”.** LGSC-4 is from a patient diagnosed with ovarian LGSC (IIIB) at the age of 66 (LGSC-4-P), followed by two separate recurrences 25 months (LGSC-4-R1) and 45 months (LGSC-4-R2) later (all 20X). (PDF 8 MB)

Additional file 5: **“LGSC-5 Case Images”.** LGSC-5 is from a patient diagnosed with LGSC (stage IIIC) at age 51 (LGSC-5-P, top) and recurrent LGSC 37 months later (LGSC-5-R, bottom; both images 100X). (PDF 5 MB)

Additional file 6: **“LGSC-6 Case Images”.** LGSC-6 is from a patient diagnosed with LGSC (stage IIIC) at 41 years old (LGSC-6-P, top) and recurrent LGSC 24 months later (LGSC-6-R, bottom; both images 100X). (PDF 5 MB)

Additional file 7: **“LGSC-8 Case Images”.** LGSC-8 is from a patient diagnosed with metastatic LGSC (stage IIIC) at the age of 33 (LGSC-8-P, top), with disease recurrence 7 months later (LGSC-8-R, bottom; both images 100X). (PDF 5 MB)

Additional file 8: **“LGSC-9 Case Images”.** LGSC-9 is from a patient diagnosed with a serous borderline tumor (stage IIIB) at age 51 (LGSC-9-P1, LGSC-9-P2, LGSC-9-R3 shown in left panels). This patient received no additional treatment after surgical resection and recurred with LGSC 100 months later (LGSC-9-R1, LGSC-9-R2, LGSC-9-R3 shown in right panels; all images 20X). (PDF 14 MB)

Additional file 9: **“LGSC-10 Case Images”.** LGSC-10 is from a patient diagnosed with bilateral ovarian LGSC (stage IV) at the age of 57 (LGSC-10-P1, LGSC-10-P2, LGSC-10-P3, LGSC-10-P4 shown in left panels), followed by disease recurrence 45 months later (LGSC-10-R1, LGSC-10-R2, LGSC-10-R3, LGSC-10-R4 shown in right panels; all images at 20X). (PDF 13 MB)

Additional file 10: **“LGSC-11 Case Images”.** LGSC-11 is from a patient diagnosed with a serous borderline tumor (stage IIIC) at 62 years (LGSC-11-P, top) followed by recurrence with LGSC 13 years later (LGSC-11-R, bottom; both images at 20X). (PDF 6 MB)

Additional file 11: **“LGSC-12 Case Images”.** LGSC-12 is from a patient diagnosed with LGSC (stage IIB) at the age of 57 (LGSC-12-P1, LGSC-12-P2, LGSC-12-P3, LGSC-12-P4 are shown). This patient was treated with etoposide, tamoxifen and anastrozole prior to recurrence with LGSC 18 months later (LGSC-12-R1, LGSC-12-R2 are shown; all images at 20X). (PDF 13 MB)

Additional file 12: **“LGSC-13 Case Images”.** LGSC-13 is from a patient diagnosed with LGSC (stage IIIB) at the age of 58 (LGSC-13-P, top), followed by recurrence with LGSC 46 months later (LGSC-13-R, bottom; both images 20X). (PDF 6 MB)

Additional file 13: **“Supplemental Methods”.** Describes additional methodological details for DNA extraction, sequencing and digital PCR. (DOCX 13 KB)

Additional file 14: **“Genes/Mutations on Ion Torrent AmpliSeq panel v1”.** Lists genes and hot spot mutations included on the Ion Torrent AmpliSeq panel. (XLSX 39 KB)

Additional file 15: **“Primer sequences for Sanger sequencing”.** Lists primer sequences used for validation of mutations by Sanger sequencing. (XLSX 46 KB)

Additional file 16: **“Primer sequences for digital PCR”.** Lists primer sequences used for validation of mutations by digital PCR. (XLSX 9 KB)

Additional file 17: **“Allele fraction of confirmed somatic mutations by Ion Torrent and MiSeq”.** The presence of a specific mutation (listed on left) in a specific tumor sample (listed at bottom) is indicated by a colored box in the corresponding position, with the shade of the box reflecting the allelic fraction as detected by **(A)** Ion Torrent or **(B)** MiSeq. Corresponding normal samples were all negative for the described mutations. (PDF 471 KB)

Additional file 18: **“Ion Torrent and MiSeq reads for true positive mutations”.** Lists the variant reads, total reads and variant frequency by sample for both Ion Torrent and MiSeq. (XLSX 32 KB)

Additional file 19: **“Stable** ***KRAS*** **,** ***BRAF*** **and** ***SMAD4*** **mutations in cases 3 and 12 by Sanger sequencing”.** Detection of the *KRAS* G12V mutation by Sanger sequencing in LGSC-3-P **(A)** and LGSC-3-R **(B)** are shown. Sanger sequencing also confirmed the presence of the *BRAF* V600E and the *SMAD4* R361G mutation in LGSC-12-P1 (***C*** and **F** respectively) and LGSC-12-R1 (**D** and **G** respectively), but not the corresponding normal sample LGSC-12-N (E and H respectively). (TIFF 1 MB)

Additional file 20: **“Digital PCR results”.** Lists the % mutant and % wildtype droplets corresponding to the digital PCR results shown in Figures 3 and 4. (XLSX 13 KB)

## Abbreviations

BCCA: BC cancer agency
BRAF: V-raf murine sarcoma viral oncogene homolog B1
ctDNA: Circulating tumor DNA
DNA: Deoxyribonucleic acid
ERK: Extracellular signal-regulated kinase
FFPE: Formalin-fixed paraffin-embedded
FGFR3: Fibroblast growth factor receptor 3
HGSC: High-grade serous carcinoma
KRAS: Kirsten rat sarcoma viral oncogene homolog
LGSC: Low-grade serous carcinoma
MAPK: Mitogen-activated kinase
MDACC: MD Anderson cancer centre
MEK: MAP kinase kinase
NRAS: Neuroblastoma RAS viral (v-ras) oncogene homolog
PCR: Polymerase chain reaction
PDGFRA: Platelet-derived growth factor receptor alpha
SBT: Serous borderline tumor
SMAD4: Mothers against decapentaplegic homolog 4
SNV: Single nucleotide variant
UHN: University health network.
