# Supplementary figures and images for: Intratumoral heterogeneity in a minority of ovarian low-grade serous carcinomas
Source: BMC Cancer. 2014 Dec 18;14:982. doi: 10.1186/1471-2407-14-982 (PMC4320586; doi:10.1186/1471-2407-14-982)

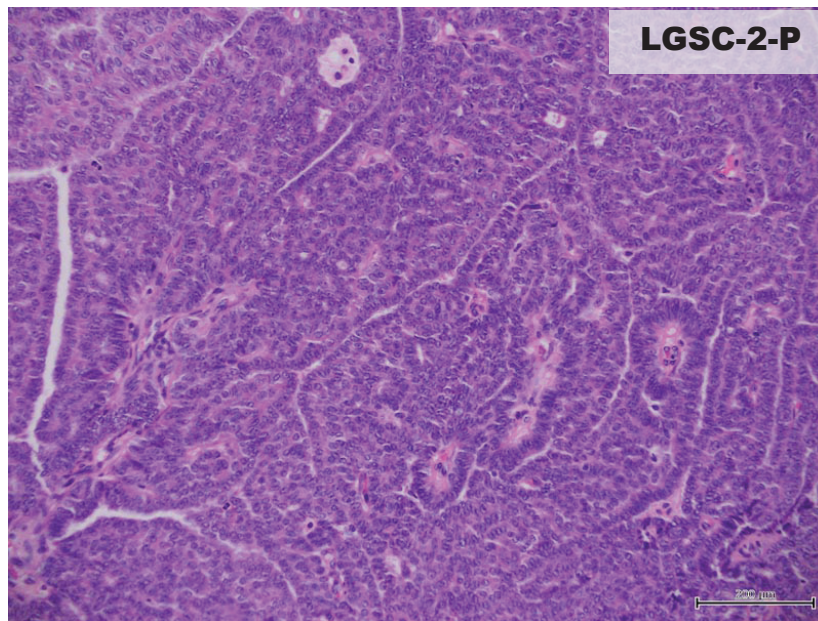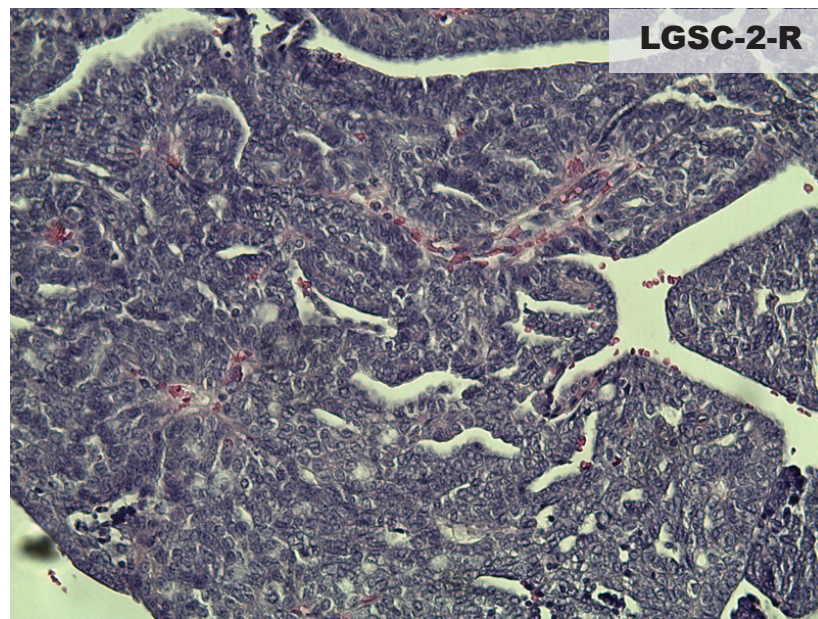

**Additional File 4. LGSC-2 Case Images.**

Supplement: Supplementary file 2 — Additional file 2: “LGSC-2 Case Images”. LGSC-2 is from a patient diagnosed with bilateral ovarian LGSC (stage IIIC) at 57 years old (LGSC-2-P, top) and metastatic LGSC 46 months after primary diagnosis (LGSC-2-R, bottom; both 20X). (PDF 6 MB) [file 12885_2014_5198_MOESM2_ESM.pdf]

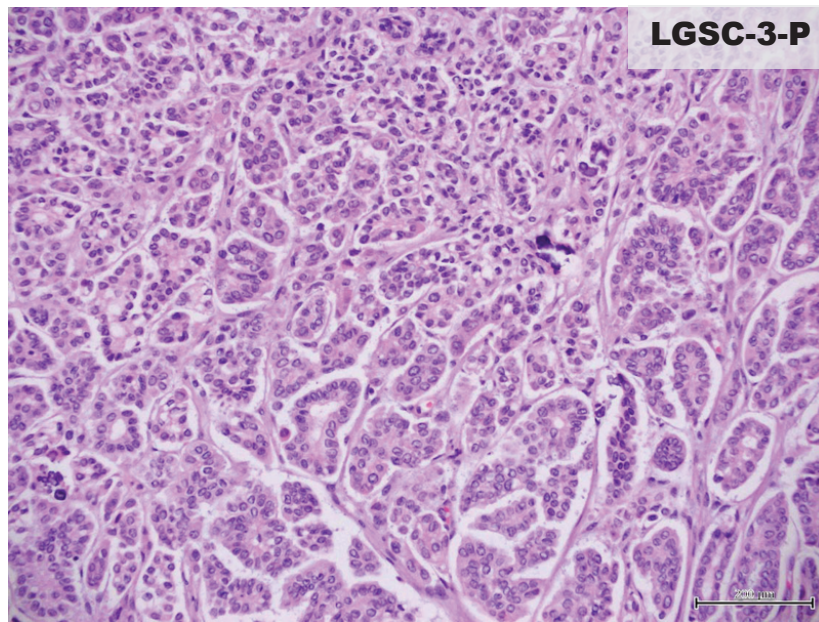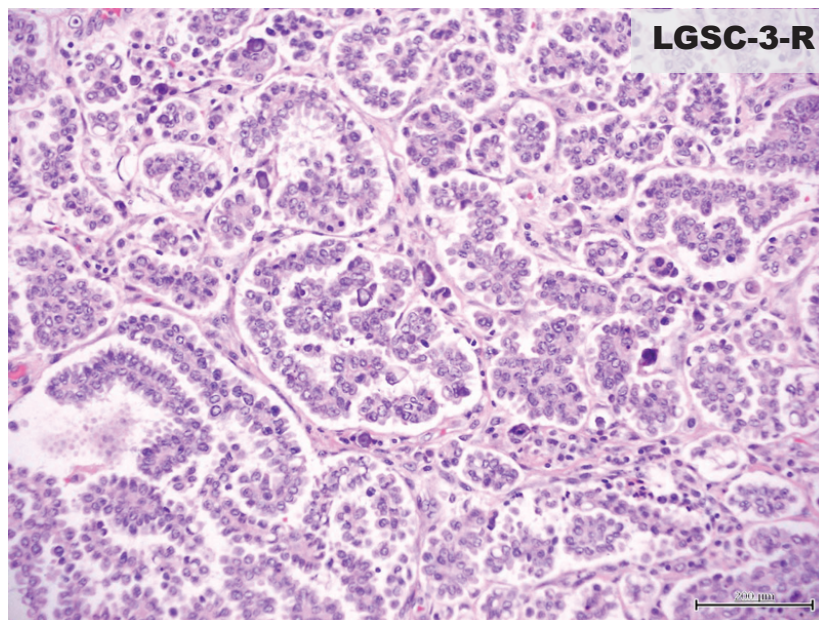

**Additional File 5. LGSC-3 Case Images.**

Supplement: Supplementary file 3 — Additional file 3: “LGSC-3 Case Images”. LGSC-3 is from a patient diagnosed with bilateral ovarian LGSC (stage IIIC) at 51 years old (LGSC-3-P, top), and recurrent LGSC 17 months later (LGSC-3-R, bottom; both 20X). (PDF 5 MB) [file 12885_2014_5198_MOESM3_ESM.pdf]

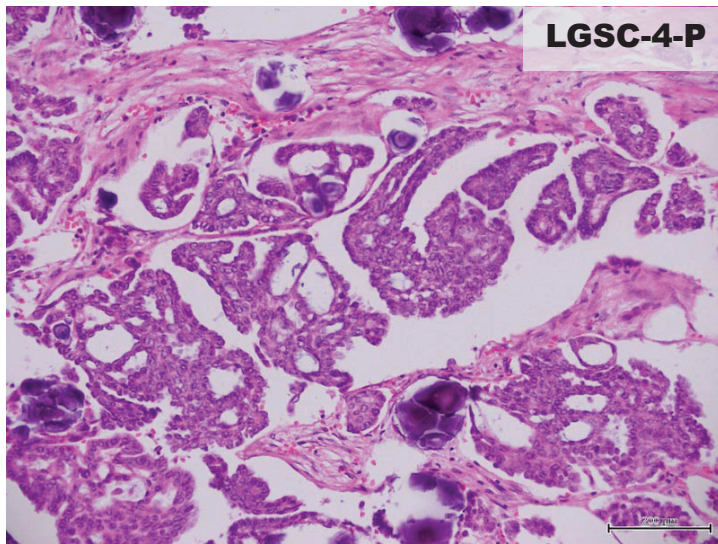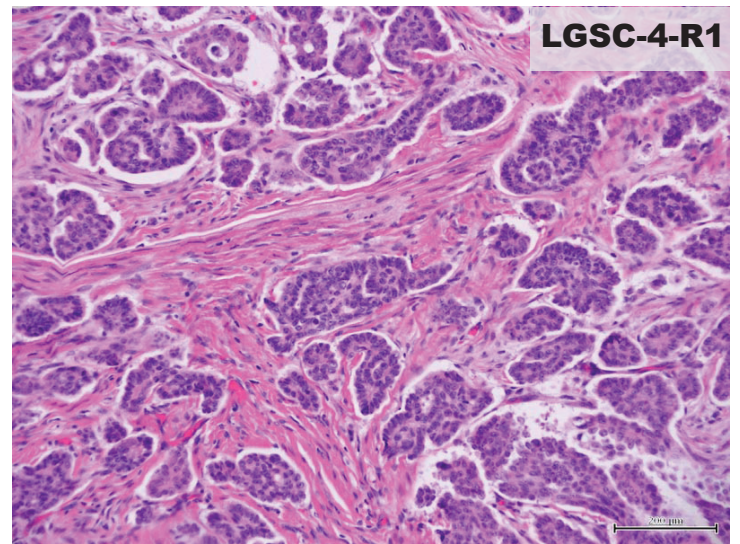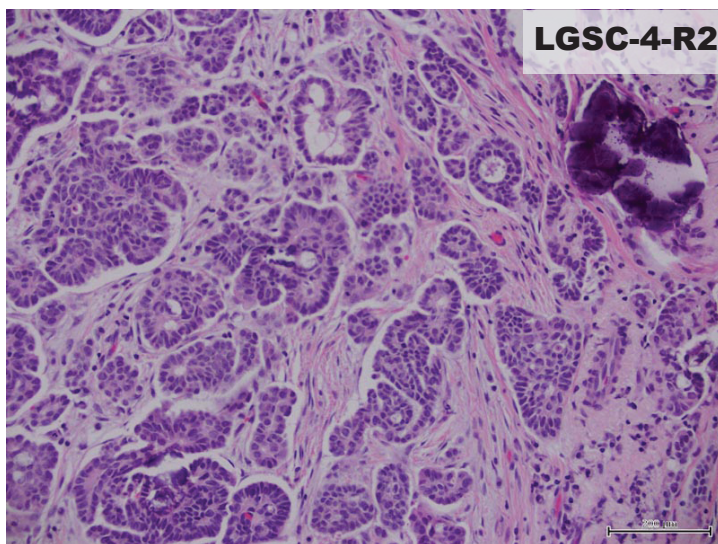

**Additional File 6. LGSC-4 Case Images.**

Supplement: Supplementary file 4 — Additional file 4: “LGSC-4 Case Images”. LGSC-4 is from a patient diagnosed with ovarian LGSC (IIIB) at the age of 66 (LGSC-4-P), followed by two separate recurrences 25 months (LGSC-4-R1) and 45 months (LGSC-4-R2) later (all 20X). (PDF 8 MB) [file 12885_2014_5198_MOESM4_ESM.pdf]

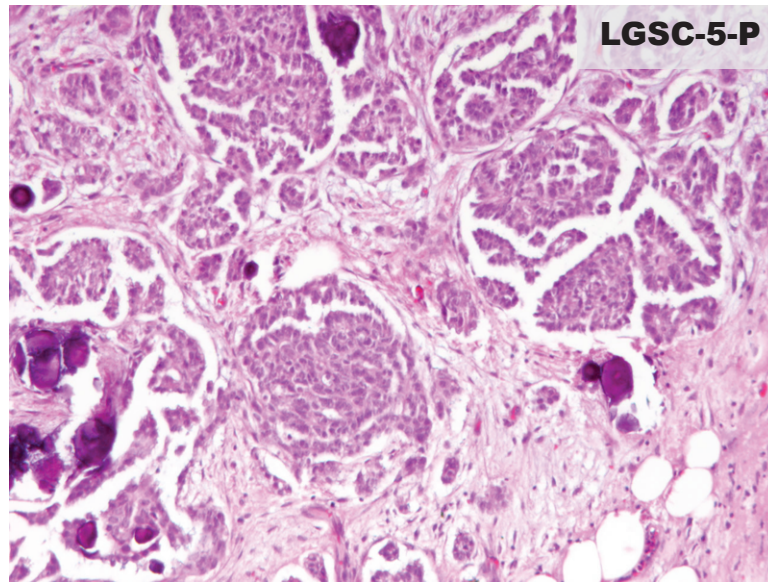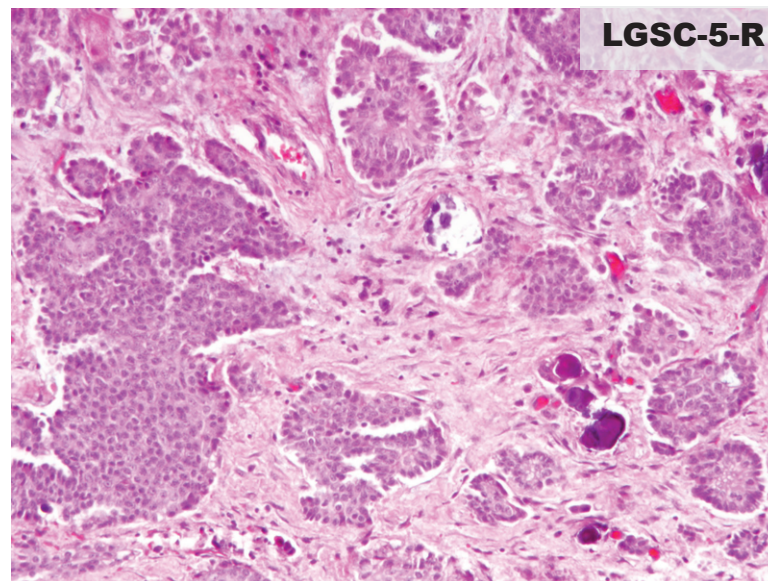

**Additional File 7. LGSC-5 Case Images.**

Supplement: Supplementary file 5 — Additional file 5: “LGSC-5 Case Images”. LGSC-5 is from a patient diagnosed with LGSC (stage IIIC) at age 51 (LGSC-5-P, top) and recurrent LGSC 37 months later (LGSC-5-R, bottom; both images 100X). (PDF 5 MB) [file 12885_2014_5198_MOESM5_ESM.pdf]

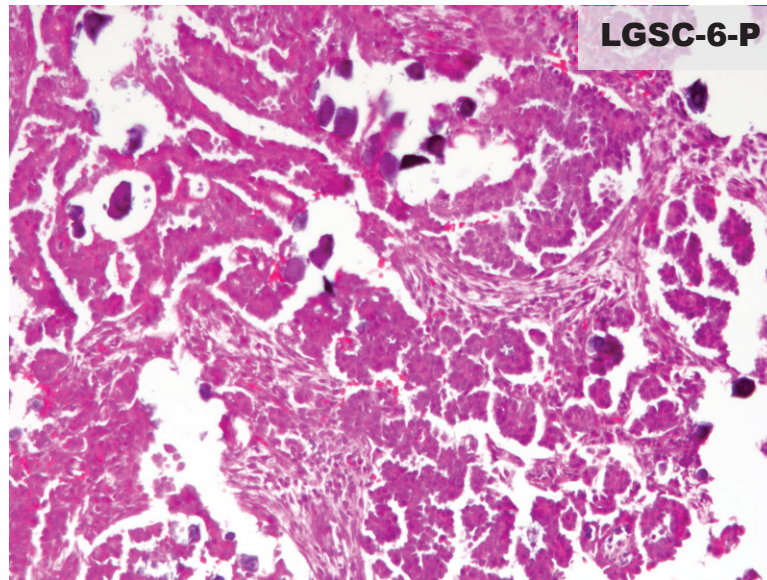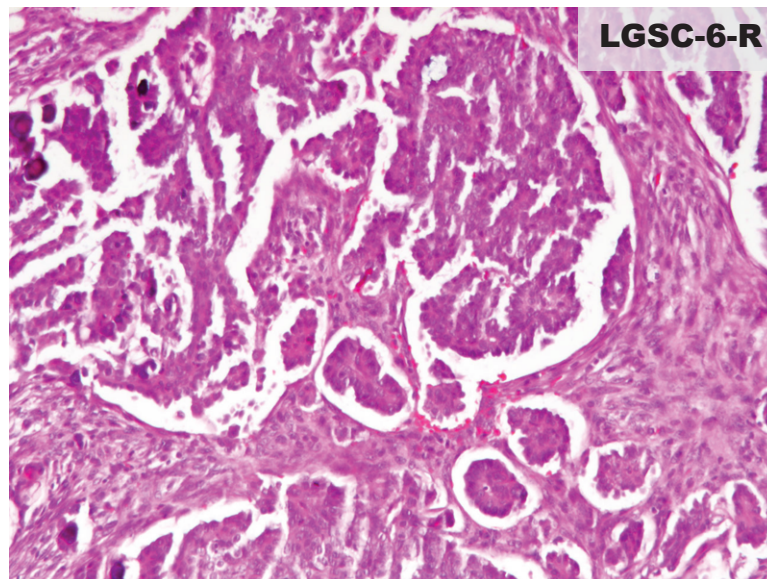

**Additional File 8. LGSC-6 Case Images.**

Supplement: Supplementary file 6 — Additional file 6: “LGSC-6 Case Images”. LGSC-6 is from a patient diagnosed with LGSC (stage IIIC) at 41 years old (LGSC-6-P, top) and recurrent LGSC 24 months later (LGSC-6-R, bottom; both images 100X). (PDF 5 MB) [file 12885_2014_5198_MOESM6_ESM.pdf]

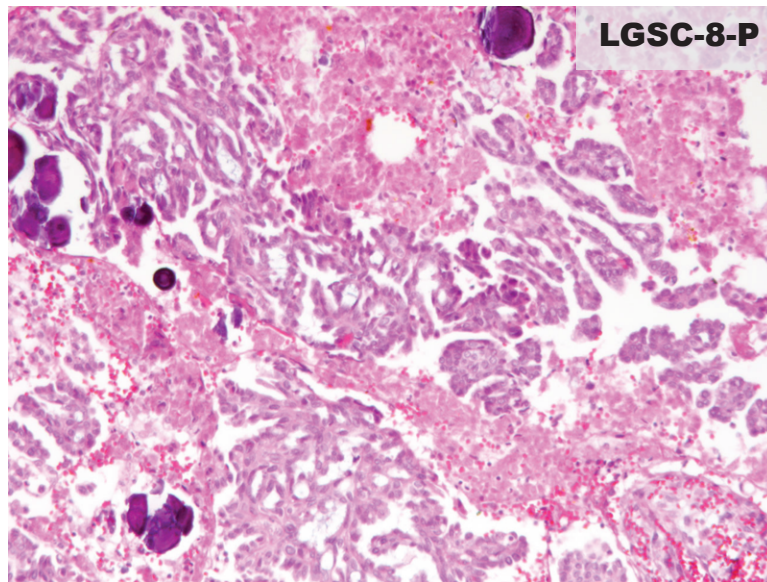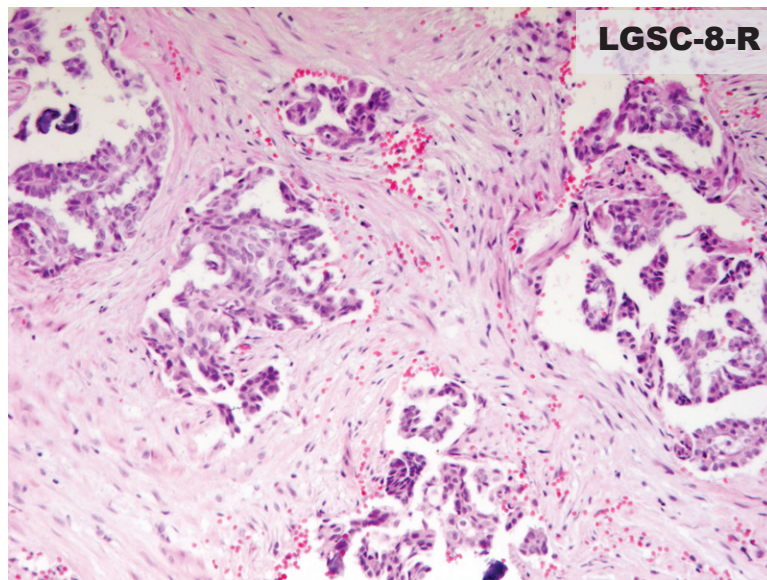

**Additional File 9. LGSC-8 Case Images.**

Supplement: Supplementary file 7 — Additional file 7: “LGSC-8 Case Images”. LGSC-8 is from a patient diagnosed with metastatic LGSC (stage IIIC) at the age of 33 (LGSC-8-P, top), with disease recurrence 7 months later (LGSC-8-R, bottom; both images 100X). (PDF 5 MB) [file 12885_2014_5198_MOESM7_ESM.pdf]

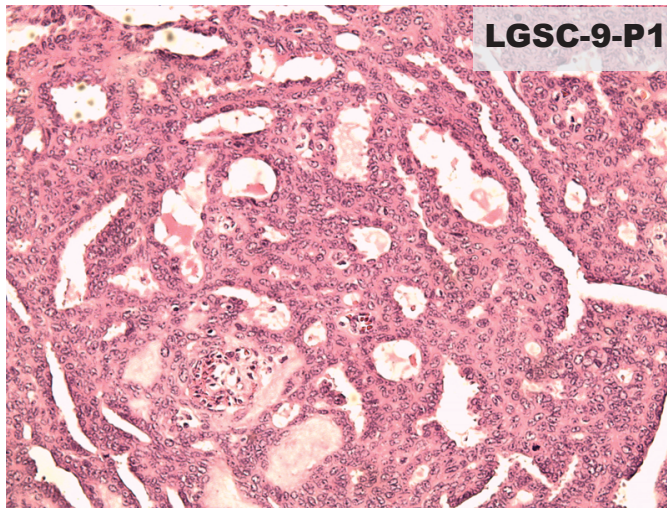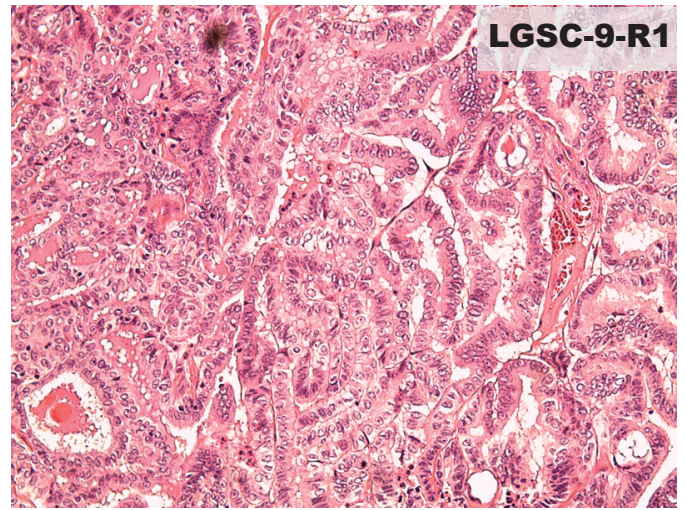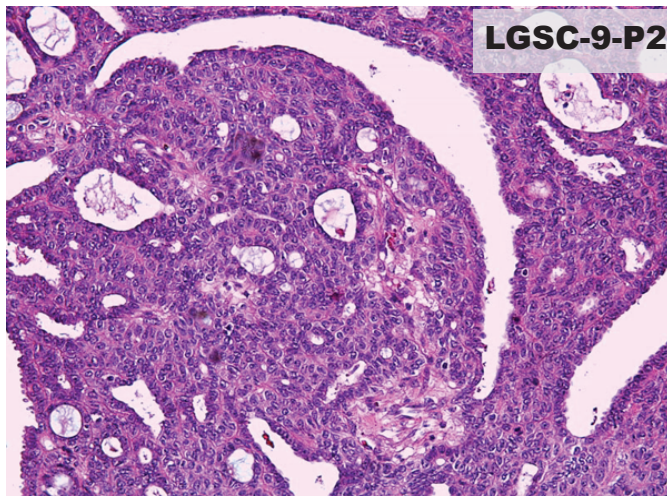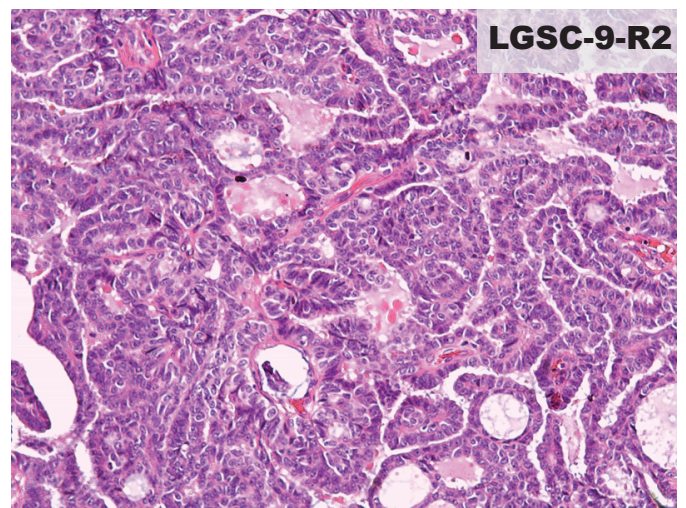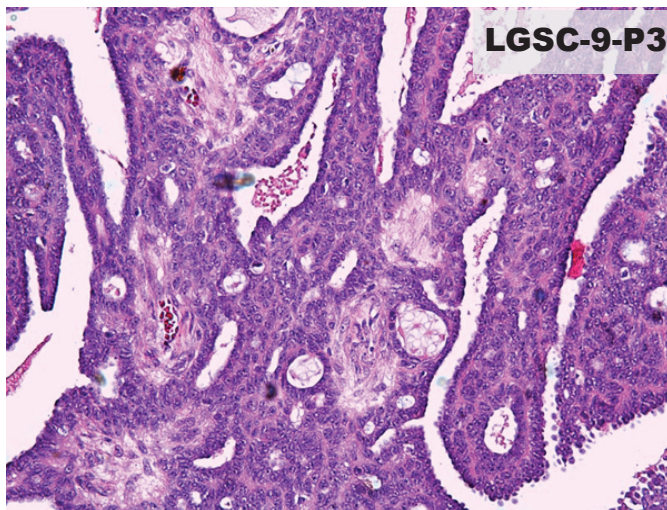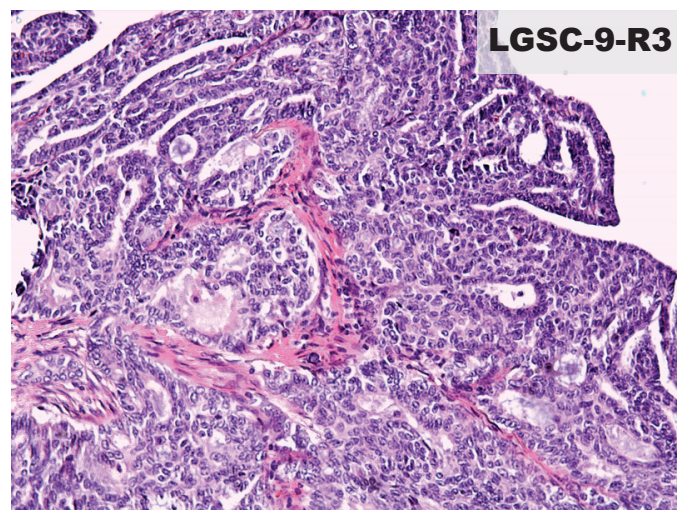

**Additional File 10. LGSC-9 Case Images.**

Supplement: Supplementary file 8 — Additional file 8: “LGSC-9 Case Images”. LGSC-9 is from a patient diagnosed with a serous borderline tumor (stage IIIB) at age 51 (LGSC-9-P1, LGSC-9-P2, LGSC-9-R3 shown in left panels). This patient received no additional treatment after surgical resection and recurred with LGSC 100 months later (LGSC-9-R1, LGSC-9-R2, LGSC-9-R3 shown in right panels; all images 20X). (PDF 14 MB) [file 12885_2014_5198_MOESM8_ESM.pdf]

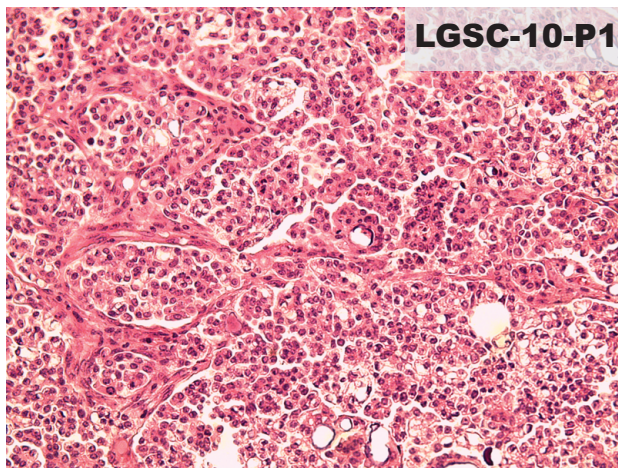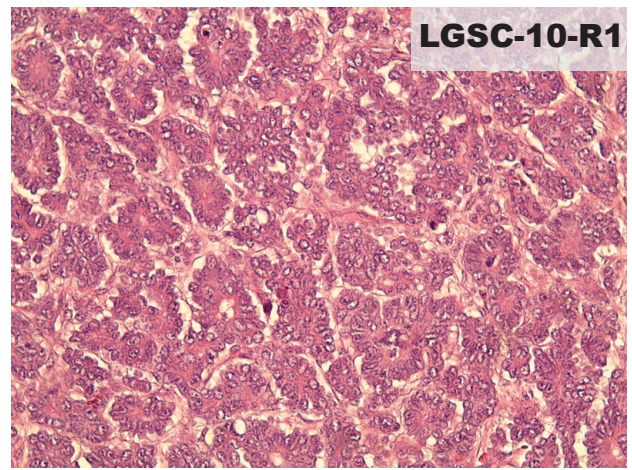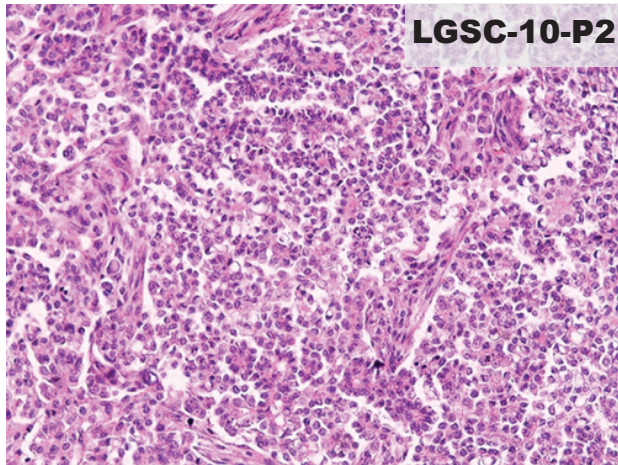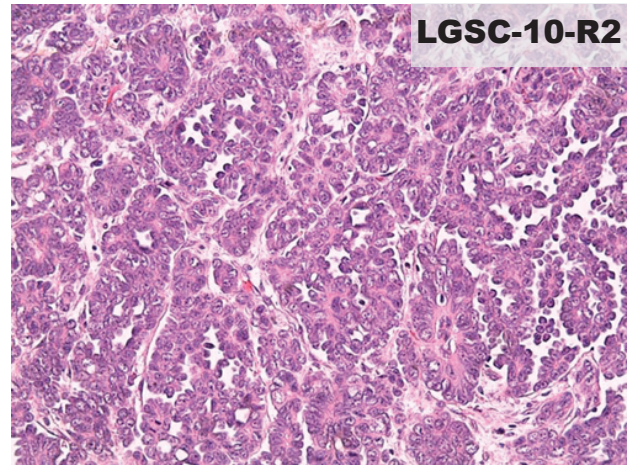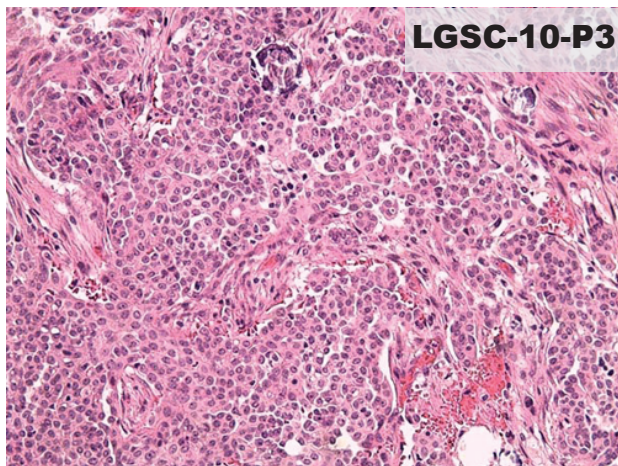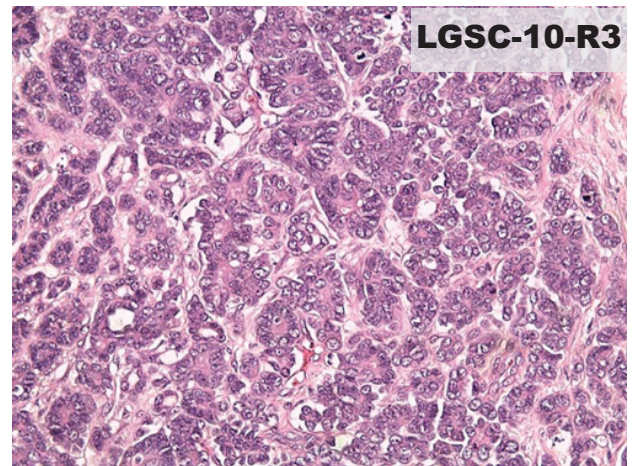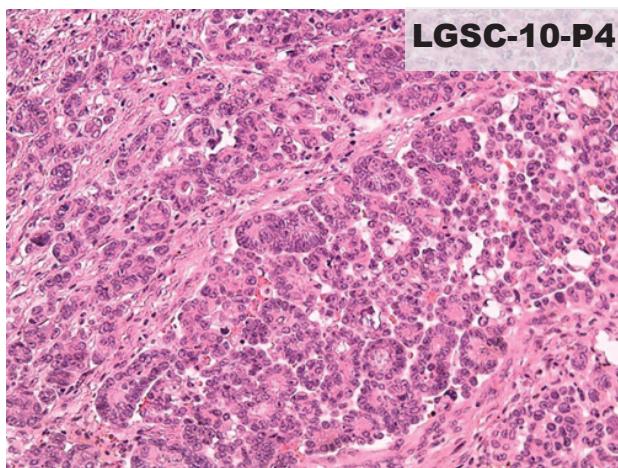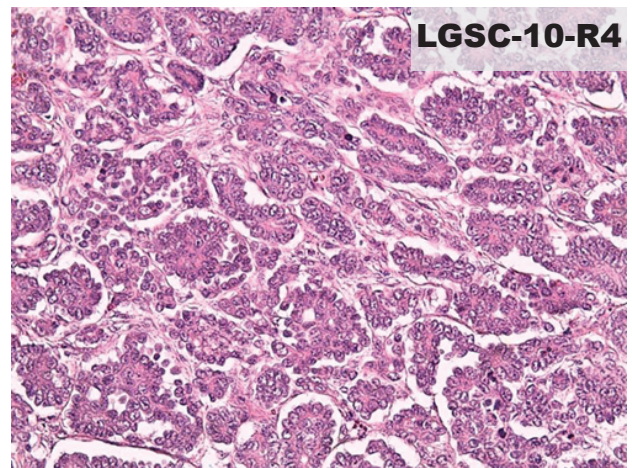

**Additional File 11. LGSC-10 Case Images.**

Supplement: Supplementary file 9 — Additional file 9: “LGSC-10 Case Images”. LGSC-10 is from a patient diagnosed with bilateral ovarian LGSC (stage IV) at the age of 57 (LGSC-10-P1, LGSC-10-P2, LGSC-10-P3, LGSC-10-P4 shown in left panels), followed by disease recurrence 45 months later (LGSC-10-R1, LGSC-10-R2, LGSC-10-R3, LGSC-10-R4 shown in right panels; all images at 20X). (PDF 13 MB) [file 12885_2014_5198_MOESM9_ESM.pdf]

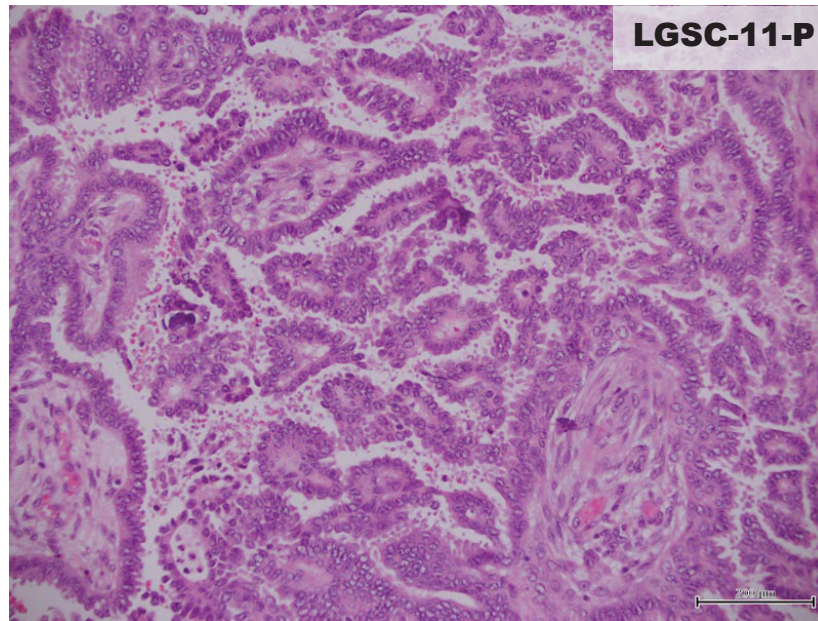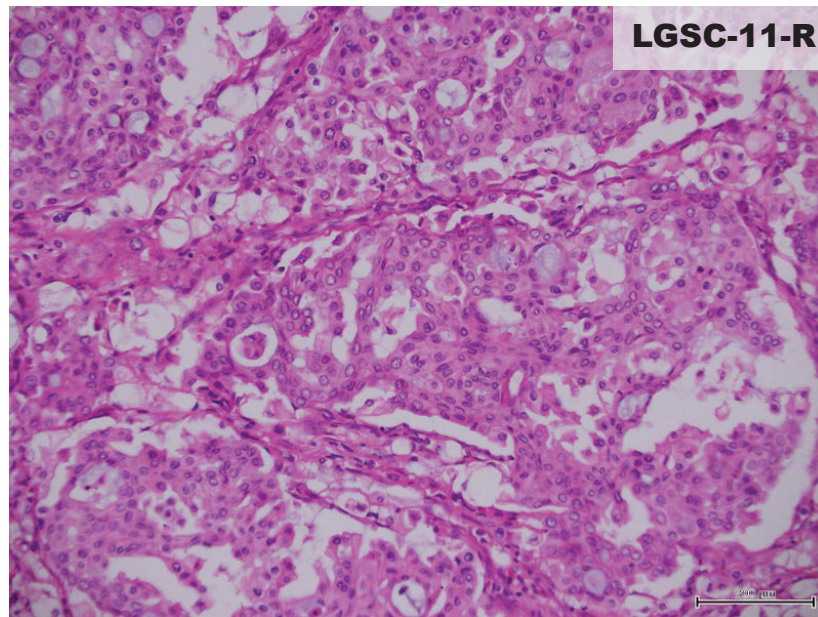

**Additional File 12. LGSC-11 Case Images.**

Supplement: Supplementary file 10 — Additional file 10: “LGSC-11 Case Images”. LGSC-11 is from a patient diagnosed with a serous borderline tumor (stage IIIC) at 62 years (LGSC-11-P, top) followed by recurrence with LGSC 13 years later (LGSC-11-R, bottom; both images at 20X). (PDF 6 MB) [file 12885_2014_5198_MOESM10_ESM.pdf]

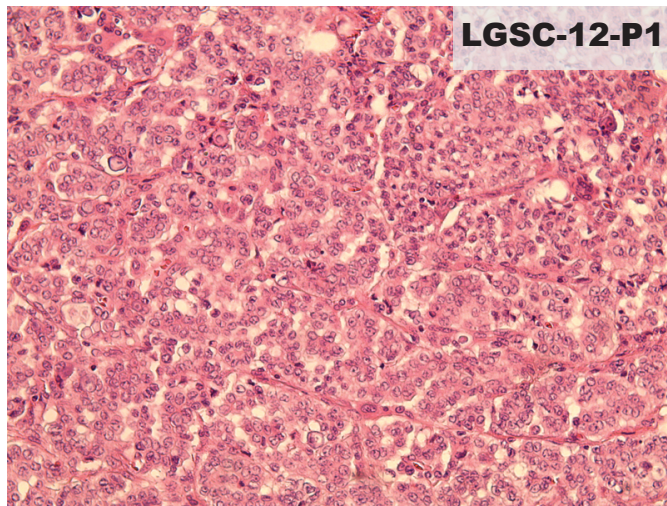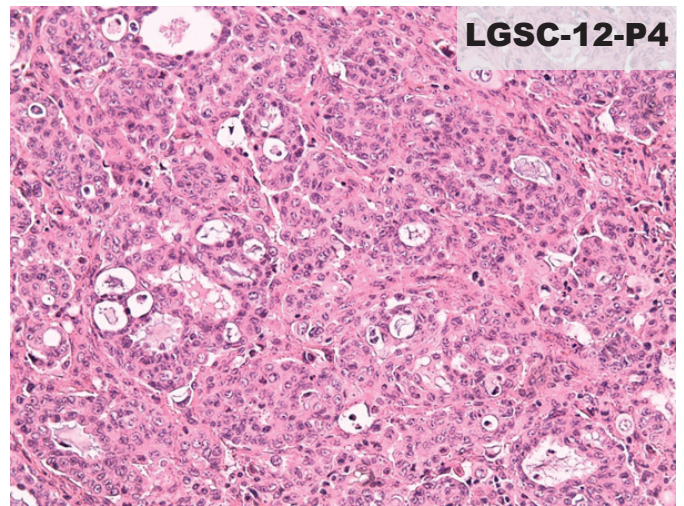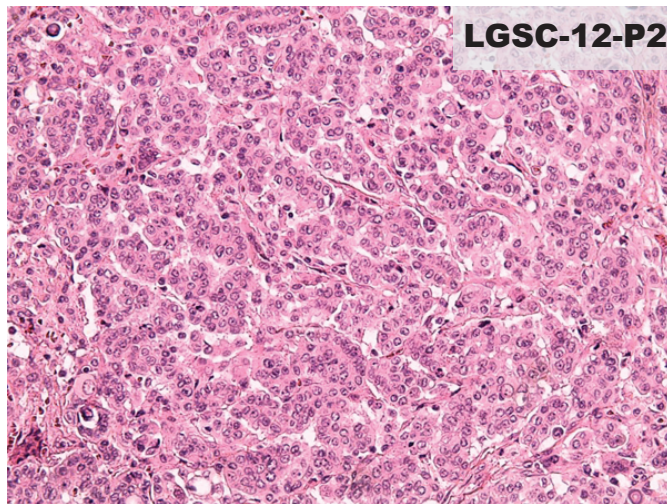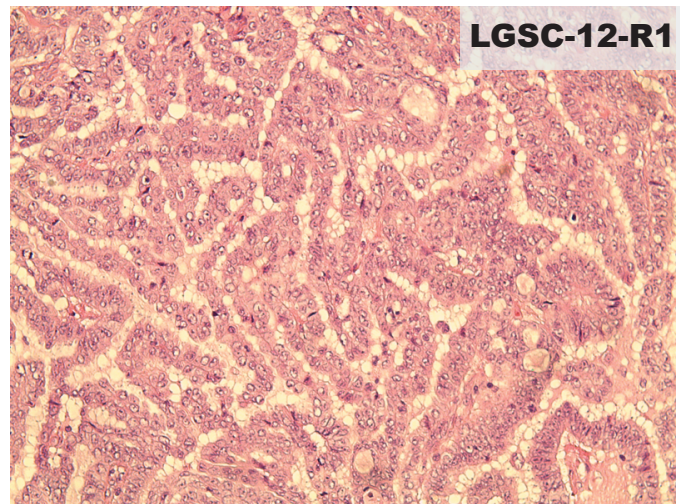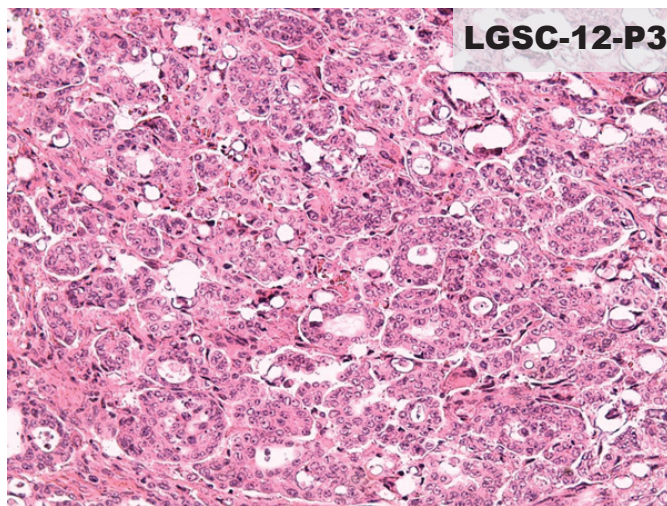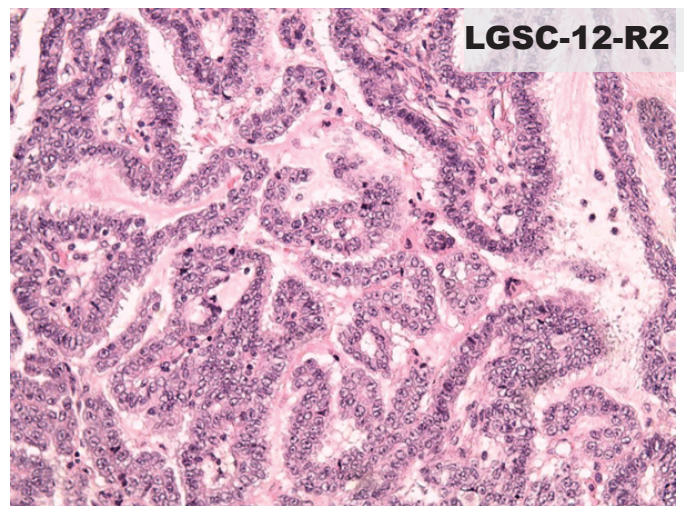

**Additional File 13. LGSC-12 Case Images.**

Supplement: Supplementary file 11 — Additional file 11: “LGSC-12 Case Images”. LGSC-12 is from a patient diagnosed with LGSC (stage IIB) at the age of 57 (LGSC-12-P1, LGSC-12-P2, LGSC-12-P3, LGSC-12-P4 are shown). This patient was treated with etoposide, tamoxifen and anastrozole prior to recurrence with LGSC 18 months later (LGSC-12-R1, LGSC-12-R2 are shown; all images at 20X). (PDF 13 MB) [file 12885_2014_5198_MOESM11_ESM.pdf]

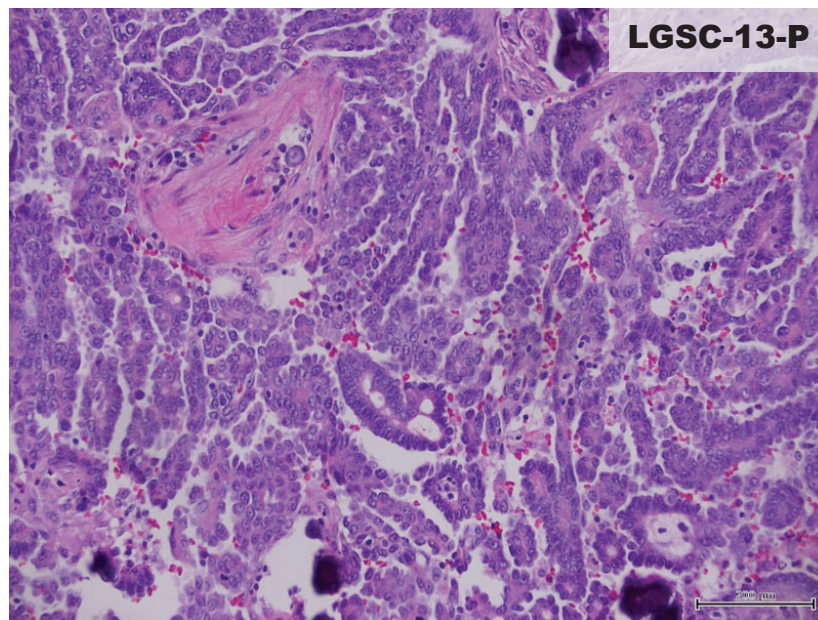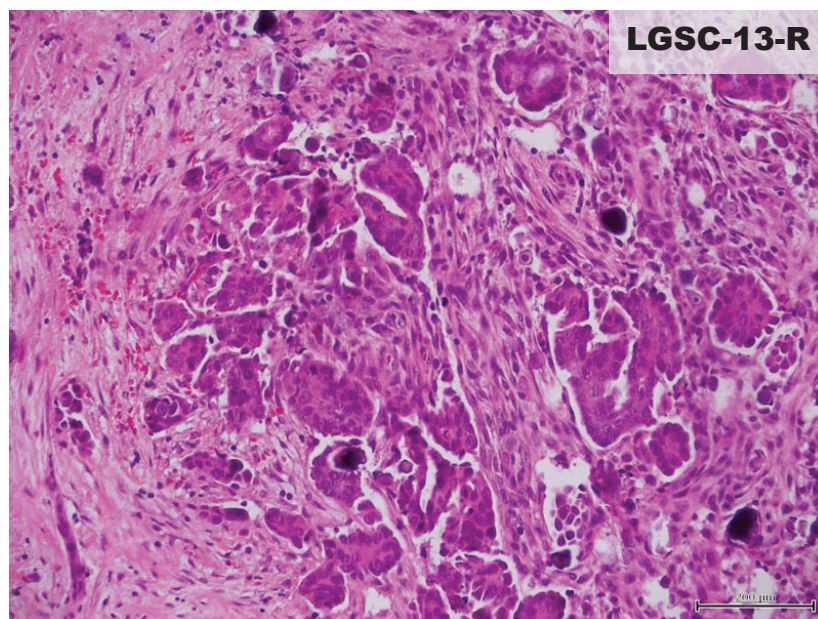

**Additional File 14. LGSC-13 Case Images.**

Supplement: Supplementary file 12 — Additional file 12: “LGSC-13 Case Images”. LGSC-13 is from a patient diagnosed with LGSC (stage IIIB) at the age of 58 (LGSC-13-P, top), followed by recurrence with LGSC 46 months later (LGSC-13-R, bottom; both images 20X). (PDF 6 MB) [file 12885_2014_5198_MOESM12_ESM.pdf]

ADDITIONAL FILE 17. FREQUENCY OF CONFIRMED SOMATIC MUTATIONS BY ION TORRENT (A) AND MISEQ (B).

A.

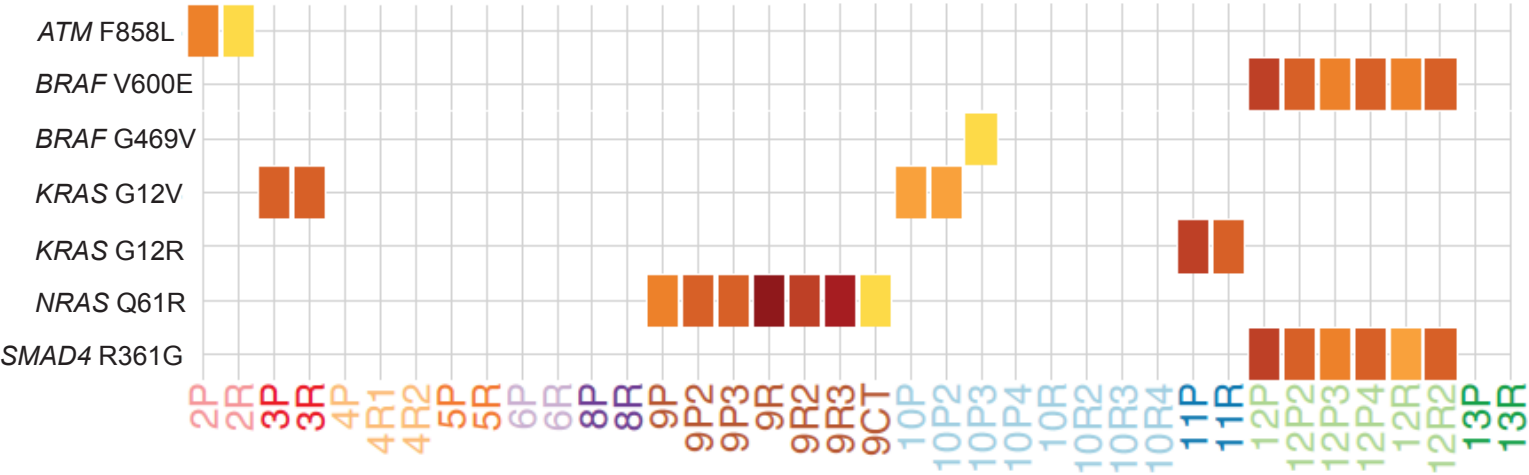

B.

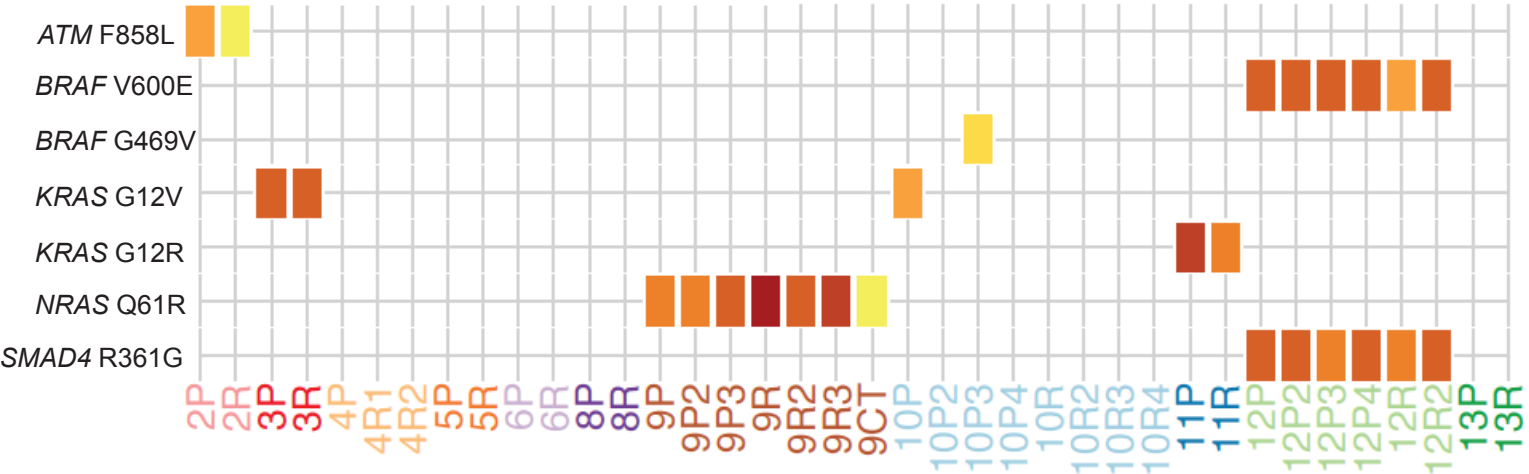

Supplement: Supplementary file 17 — Additional file 17: “Allele fraction of confirmed somatic mutations by Ion Torrent and MiSeq”. The presence of a specific mutation (listed on left) in a specific tumor sample (listed at bottom) is indicated by a colored box in the corresponding position, with the shade of the box reflecting the allelic fraction as detected by (A) Ion Torrent or (B) MiSeq. Corresponding normal samples were all negative for the described mutations. (PDF 471 KB) [file 12885_2014_5198_MOESM17_ESM.pdf]

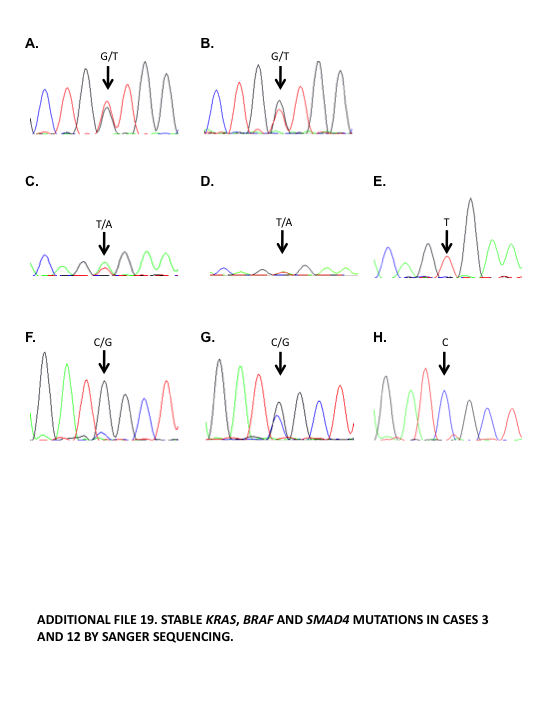

Supplement: Supplementary file 19 — Additional file 19: “Stable KRAS , BRAF and SMAD4 mutations in cases 3 and 12 by Sanger sequencing”. Detection of the KRAS G12V mutation by Sanger sequencing in LGSC-3-P (A) and LGSC-3-R (B) are shown. Sanger sequencing also confirmed the presence of the BRAF V600E and the SMAD4 R361G mutation in LGSC-12-P1 (C and F respectively) and LGSC-12-R1 (D and G respectively), but not the corresponding normal sample LGSC-12-N (E and H respectively). (TIFF 1 MB) [file 12885_2014_5198_MOESM19_ESM.tiff]
