# Supplementary material for: Intratumoral heterogeneity in a minority of ovarian low-grade serous carcinomas
Source: BMC Cancer. 2014 Dec 18;14:982. doi: 10.1186/1471-2407-14-982 (PMC4320586; doi:10.1186/1471-2407-14-982)
Supplement: Supplementary file 13 — Additional file 13: “Supplemental Methods”. Describes additional methodological details for DNA extraction, sequencing and digital PCR. (DOCX 13 KB) [file 12885_2014_5198_MOESM13_ESM.docx]

**ADDITIONAL FILE 1. SUPPLEMENTAL METHODS.**

***DNA extraction.*** Tumor and normal DNA from FFPE blocks/unstained sections was extracted using the Qiagen QIAamp DNA FFPE extraction kit (Qiagen, Toronto/Ont/CAN). DNA was eluted in Buffer ATE and quantified using the Qubit Fluorometer (Life Technologies, CA/USA).

***Ion Torrent AmpliSeq Cancer Hotspot sequencing.*** The Ion Torrent AmpliSeq^TM^ Cancer Hotspot Panel Version 1 (Life Technologies, Grand Island/NY/USA; version Jan-2012) covers 739 mutations in 46 genes previously implicated in cancer (PMID: 21776081). In brief, 10ng of DNA is amplified with a cancer hotspot primer pool, barcodes and adapters are ligated to samples, and final libraries are quality controlled on an Agilent Bioanalyzer (Agilent Technologies, Santa Clara/CA/USA). Oil emulsion PCR was performed using the Ion One-touch machine with the 200 Template Kit. The amplicon enriched Ion Sphere^TM^ Particles beads were then purified using the Invitrogen Dynabeads MyOne^TM^ Streptavidin C1 (Life Technologies, Grand Island/NY/USA). Three barcoded libraries were pooled for loading onto each Ion 316^TM^ chip followed by sequencing on the Ion PGM^TM^ sequencer. The plasma ctDNA library was sequenced on a single Ion 316^TM^ chip.

***Fluidigm-MiSeq Targeted Sequencing Validation.*** Primers for Fluidigm amplification were designed for each selected SNV, using Primer 3 with minimum 150bp to 200bp amplicon size. Amplicon-specific primers included Fluidigm sequencing adaptors CS1 (ACACTGACGACATGGTTCTACA) and CS2 (TACGGTAGCAGAGACTTGGTCT), and were obtained from IDT (Integrated DNA Technologies, Coralville/Iowa/USA), as well as 48 barcodes containing Illumina cluster adapter sequences. Custom LNA (Locked Nucleic Acid) sequencing primers for CS1, CS2 and the index read were obtained from Exiqon (Woburn/MA/USA). All amplification protocols were adapted from Fluidigm protocols and TAM-Seq as previously described (PMID: 22649089). In brief, a DNA pre-amplification step with the pooled primer library was performed, then loaded onto a Fluidigm 48X48 Access Array chip for library amplification. Barcodes for a single index read on the Illumina Miseq were amplified onto the targeted amplicons post Fluidigm harvest, quantified by Qubit then pooled at equal concentrations. The pooled library was quantified by KAPA qPCR (KAPA Biosystems, Woburn/MA/USA), diluted and denatured to 10pM library as per the Illumina MiSeq protocol, then sequenced using the 300cycle V2 MiSeq kit. Sequence reads were aligned using the mem algorithm of BWA v0.7.4 to a reference database containing only the targeted loci. The aligned reads had a mean length of 141bp (standard deviation 31bp). Low quality reads with mapping quality <20 were removed from further analysis. The targeted positions had a mean coverage of 17,030 reads (standard deviation 9,345), excluding low quality bases (base-quality <10).

***Raindance Raindrop digital PCR assay.*** In brief, 25uL reactions were used with Applied Biosystems 2X genotyping master mix, 40X primer probe, 10X drop stabilizer, and 20ng of DNA. Oil droplets were generated on the Raindrop Source chip instrument, then amplified on an MJ Tetrad thermocycler using the following conditions: 95^o^C for 10min, 45 cycles of 95^o^C for 15sec, 60^o^C for 45sec (slow ramp speed 0.5^o^C/second), final 98^o^C for 10min, hold at 4^o^C. Single fluorescent droplets were detected on the Raindance Raindrop Sense chip instrument, then analyzed using the Raindance Raindrop Analyst software V2 to count wild type and mutant drops and to prepare output graphs.
